# *egr1* and *egr4* regulate zebrafish renal regeneration by promoting *foxm1* expression

**DOI:** 10.1093/jmcb/mjaf026

**Published:** 2025-08-15

**Authors:** Xian He, Yuhua Sun

**Affiliations:** Key Laboratory of Breeding Biotechnology and Sustainable Aquaculture, Institute of Hydrobiology, Chinese Academy of Sciences, Wuhan 430072, China; The Innovation of Seed Design, Chinese Academy of Sciences, Wuhan 430072, China; College of Advanced Agricultural Sciences, University of Chinese Academy of Sciences, Beijing 100049, China; Key Laboratory of Breeding Biotechnology and Sustainable Aquaculture, Institute of Hydrobiology, Chinese Academy of Sciences, Wuhan 430072, China; The Innovation of Seed Design, Chinese Academy of Sciences, Wuhan 430072, China; College of Advanced Agricultural Sciences, University of Chinese Academy of Sciences, Beijing 100049, China; Hubei Hongshan Laboratory, Wuhan 430070, China

**Keywords:** early growth response factors, renal regeneration, proliferation, apoptosis, foxm1, AKI

## Abstract

Early growth response (Egr) factors are involved in tissue development and repair. However, few studies have focused on the role of *egr* genes in renal regeneration after acute kidney injury (AKI) and the underlying mechanisms. In this study, we observed that *egr1* and *egr4* were sharply upregulated in wild type zebrafish at 1 day post-injury by gentamicin. Further experiments with *egr1* and *egr4* mutants showed that *egr1* and *egr4* were involved in zebrafish renal regeneration after AKI by regulating the proliferation and apoptosis of tubular cells. *foxm1* is expressed in injured kidneys and involved in kidney repair. Loss of *foxm1* inhibited zebrafish renal regeneration by decreasing the proliferation and increasing the apoptosis of tubular cells. Moreover, Egr1 and Egr4 promoted *foxm1* expression by directly binding to the *foxm1* promoter, thus regulating renal regeneration. Our results revealed that the rapid and transient induction of *egr1* and *egr4* after AKI exerts a renoprotective role through upregulating *foxm1* to facilitate kidney regeneration. Therefore, the *egr1*/*egr4*–*foxm1* regulatory axis holds a therapeutic potential for the treatment of AKI.

## Introduction

In recent years, the morbidity of kidney diseases has increased, with a consequent dramatic impact on people's health, resulting in high mortality among patients (Chertow et al., 2005). Acute kidney injury (AKI) is a severe renal disease consisting of a sudden loss of renal function caused by nephrotoxins, infections, or physical injuries. It is associated with high risks of developing chronic kidney disease and end-stage renal disease (Abd ElHafeez et al., 2017). AKI affects most segments of the nephron, with proximal tubules (PTs) being the most vulnerable to injury (Izzedine and Perazella, 2017). Moreover, AKI induces apoptosis or necrosis of renal tubular epithelial cells, especially in PTs (He et al., 2017).

Tubular epithelial cells change their characteristics in response to injury and the subsequent regeneration process; these changes are the key contributors in maintaining renal homeostasis and tubular restoration (Kumar 2018; Qi and Yang, 2018; He et al., 2025). Surviving resident tubular cells rebuild tubules after injury through regulating cell dedifferentiation, proliferation, and redifferentiation to restore nephron structural integrity and function (Agarwal et al., 2016; Andrade et al., 2018). The gene expression pattern of nephron cells also dramatically changes to coordinate the state of tubular cells after injury.

Early growth response (*egr*) genes belong to the family of DNA-binding transcription factors and are rapidly and transiently induced in tissues by injury and other stimuli (Tarcic et al., 2011; He et al., 2025), thus called ‘immediate early response’ factors. *egr* genes are conserved in vertebrates and widely expressed in almost all tissues. They participate in tissue development, injury response, and regeneration through regulating physiological processes such as cell proliferation, apoptosis, invasion, and differentiation (Zins et al., 2014; Wang et al., 2021; Chen et al., 2022). Studies revealed that Egr factors directly bind to the chromatin to activate the expression of genes required for tissue regeneration after injury (Gehrke et al., 2019; Chen et al., 2022). Many *egr* genes are paralogs, such as *egr1* and *egr4*. The functions of these genes are complementary in organisms and dynamically regulated to compensate for the loss of other genes. Previous research on the regeneration of adult mouse kidney showed that the increased *egr1* expression enhances kidney repair by directly binding to the downstream genes after injury (Chen et al., 2022). Although the involvement of *egr1* in mammalian kidney repair has been reported, its role in kidney diseases is not yet fully understood, while the study of *egr4* on renal regeneration is still not available. Moreover, neither *egr1* nor *egr4* was reported to be involved in the modulation of zebrafish renal regeneration after AKI.

Forkhead box protein M1 (FoxM1) is a transcription factor in the forkhead box family, which includes evolutionarily conserved regulators possessing DNA-binding activity through the forkhead box or winged helix domain (Wierstra and Alves, 2007). Previous studies indicated that *foxm1* is involved in tissue regeneration by regulating cell proliferation, apoptosis, differentiation, migration, invasion, and oxidative stress (Laoukili et al., 2005; Zu et al., 2019; Sinha et al., 2020). Loss of *foxm1* leads to defects in cell mitosis and induces apoptosis (Blagosklonny et al., 2009), while *foxm1* overexpression causes unlimited cell cycle activity and excessive cell proliferation (Kim et al., 2006). Thus, FoxM1 signaling plays critical roles in organ development and regeneration by sustaining the balance between cell proliferation and apoptosis.

In this study, we investigated the role of *egr1* and *egr4* in zebrafish renal regeneration in response to AKI. Our results demonstrated that *egr1* and *egr4* regulate cell proliferation and apoptosis by promoting *foxm1* expression in regenerating kidney, providing new insight into kidney repair by the surviving tubular cells.

## Results

### egr1 and egr4 are upregulated transiently after renal injury by gentamicin

**Figure 1:**
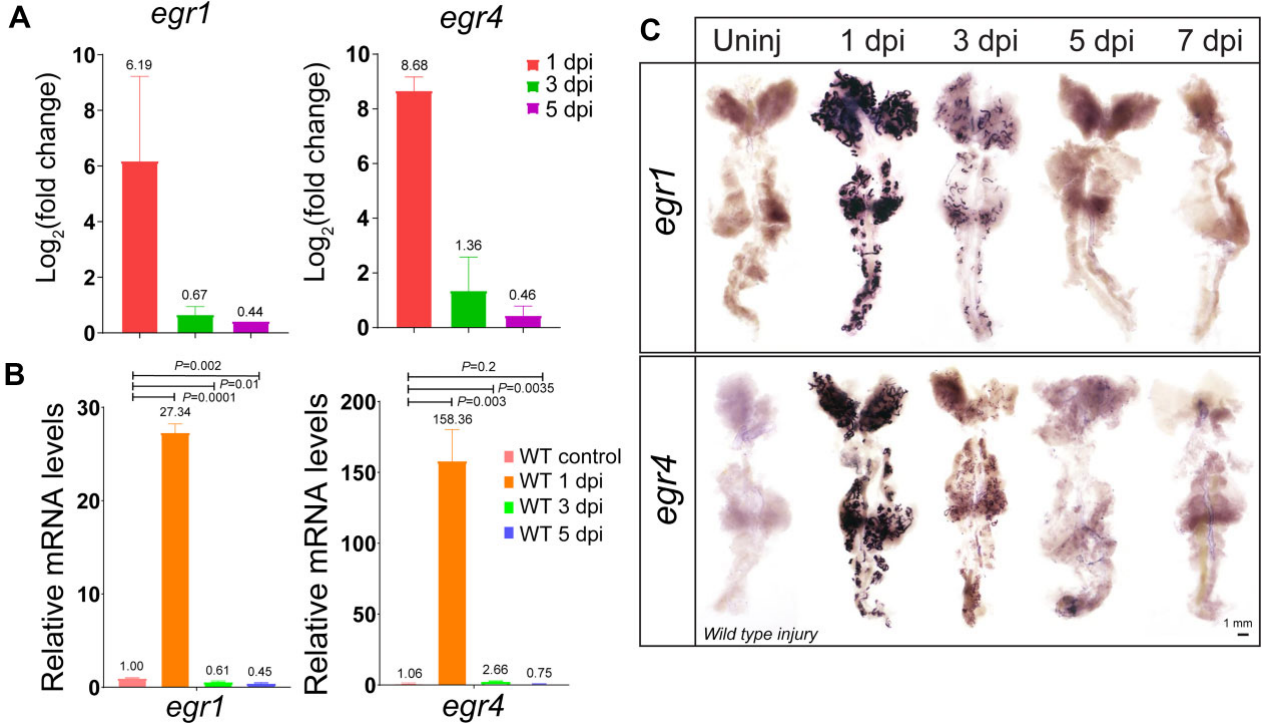
*egr1* and *egr4* are rapidly and transiently upregulated after AKI by gentamicin. (**A**) RNA-seq data showing the transient upregulation of *egr1* and *egr4* at the indicated time points. (**B**) qPCR results showing the dynamic expression of *egr1* and *egr4* at the indicated time points after AKI. Both *egr1* and *egr4* responded to kidney injury immediately and sharply at 1 dpi, and then the expression gradually decreased to normal. (**C**) WISH images showing the dynamic expression of *egr1* and *egr4* at the indicated time points.

AKI was induced by intraperitoneal injection of gentamicin into the 5-month-old adult zebrafish (Kamei et al., 2015). Zebrafish kidneys were collected at different time points, i.e. uninjured, 1 day post-injury (1 dpi), 3 dpi, and 5 dpi. RNA sequencing (RNA-seq) was performed to study the changes in gene expression, and the differentially expressed genes (DEGs) during renal regeneration were determined with |log_2_(fold change)|>1 and *q*<0.05 (Supplementary File 2; the raw data have been uploaded to the National Genomics Data Center, with the accession number CRA014881). Gene ontology (GO) enrichment analysis revealed significantly upregulated expression of ‘immediate early response’ genes at 1 dpi (Supplementary Figure S1A). In particular, *egr4* and *egr1* were ranked 2nd and 50th among the top 50 upregulated DEGs at 1 dpi (Supplementary Figure S1B). Notably, the log_2_(fold change) values of *egr1* and *egr4* at 1 dpi were much higher than those at 3 dpi or 5 dpi (Figure 1A), suggesting that *egr1* and *egr4* are important during early stages of renal regeneration.

Quantitative real-time polymerase chain reaction (qPCR) analysis of *egr1* and *egr4* mRNA levels in wild type (WT) kidneys at 1 dpi, 3 dpi, and 5 dpi confirmed that the expression of *egr1* and *egr4* was significantly upregulated at 1 dpi and dramatically reduced at 3 dpi in the injured kidneys (Figure 1B). Next, whole-mount *in situ* hybridization (WISH) was performed with specific RNA probes on the kidney samples at different time points (uninjured, 1 dpi, 3 dpi, 5 dpi, and 7 dpi). The expression of *egr1* and *erg4* in tubules was highest at 1 dpi and then gradually disappeared (Figure 1C), while the expression of the proximal convoluted tubule marker gene *slc20a1a* and the proximal straight tubule marker gene *trpm7* was barely detected at 1 dpi but gradually increased at 3 dpi, and the expression of the distal early marker gene *slc12a1* and the distal late marker gene *slc12a3* was stable in all samples (Supplementary Figure S1C). Furthermore, immunofluorescence staining for Pax2a, a marker for dedifferentiated renal PT cells and progenitors (Buzhor et al., 2013; Lazzeri et al., 2018; Liu et al., 2023) revealed that tubules underwent serious damage from 1 dpi to 3 dpi and recovered by Pax2a-positive progenitor cells increasing after 3 dpi (Supplementary Figure S1D). These results indicate that gentamicin could induce a severe damage in zebrafish kidney and nephron function loss, as well as a transient expression of *egr1* and *egr4* in response to AKI to regulate renal regeneration.

### Construction of egr1 and egr4 mutants

**Figure 2:**
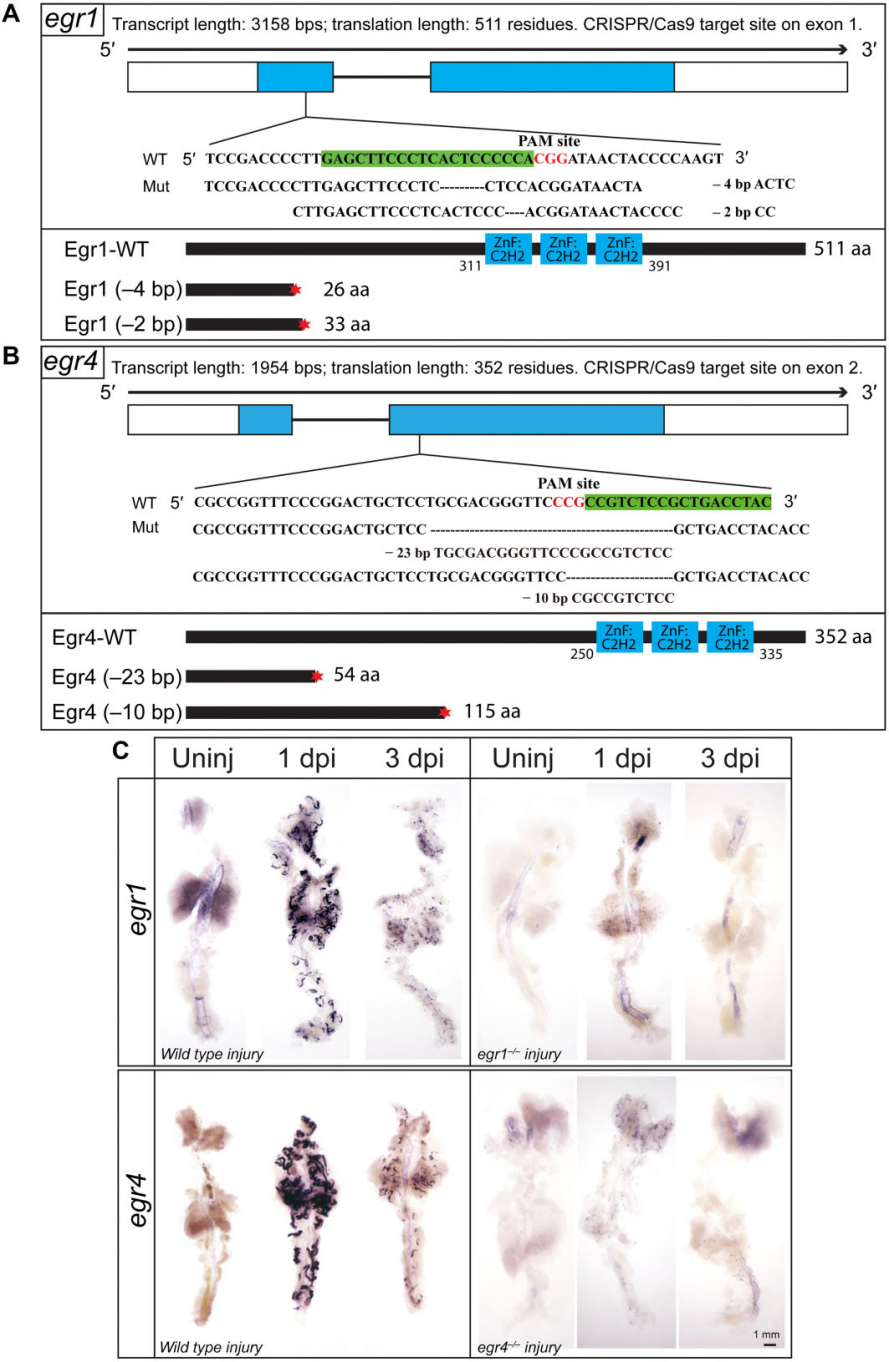
*egr1* and *egr4* mutant construction by CRISPR/Cas9. (**A**) Upper: the Cas9 target site of *egr1* was designed within the exon 1, and –2 bp and –4 bp mutant alleles were obtained. Lower: the cartoon of the full-length and respective truncated Egr1 proteins of 511 aa, 26 aa, and 33 aa, respectively. (**B**) Upper: the Cas9 target site of *egr4* on exon 2 to obtain 10 bp and 23 bp deletions. Lower: the cartoon of the full-length and truncated Egr4 proteins of 352 all, 54 aa, and 115 aa, respectively. (**C**) WISH images of *egr1* and *egr4* in wild type, *egr1*^–/–^, and *egr4*^–/–^ zebrafish. The expression of *egr1* and *egr4* was strongest at 1 dpi and weaker at 3 dpi in wild type injured kidneys but undetectable in mutant kidneys.

The function of *egr1* and *egr4* in renal regeneration following AKI was further investigated by the construction of *egr1* and *egr4* mutants using CRISPR/Cas9. Two alleles were maintained for each, i.e. –2 bp and –4 bp for *egr1* mutant and –10 bp and –23 bp for *egr4* mutant, respectively (Figure 2A and B; Supplementary Figure S2). Blast analysis of the translated proteins indicated that each mutant was prematurely terminated in two proteins, the *egr1* mutant allele proteins at 26 and 33 amino acids (aa) and the *egr4* mutant proteins at 54 and 115 aa (Figure 2A and B). *egr1* and *egr4* mutant homozygous offsprings survived into adulthood with normal phenotype and reproduction, and thus were used for subsequent kidney injury experiments. WISH results demonstrated that *egr1* and *erg4* were barely detected in *egr1*^–^^/^^–^ and *egr4*^–/–^ kidneys (either uninjured, at 1 dpi, or at 3 dpi) compared to the wild type group (Figure 2C), confirming that *egr1* and *egr4* were knocked out.

### Loss of egr1 or egr4 blocks renal regeneration

**Figure 3:**
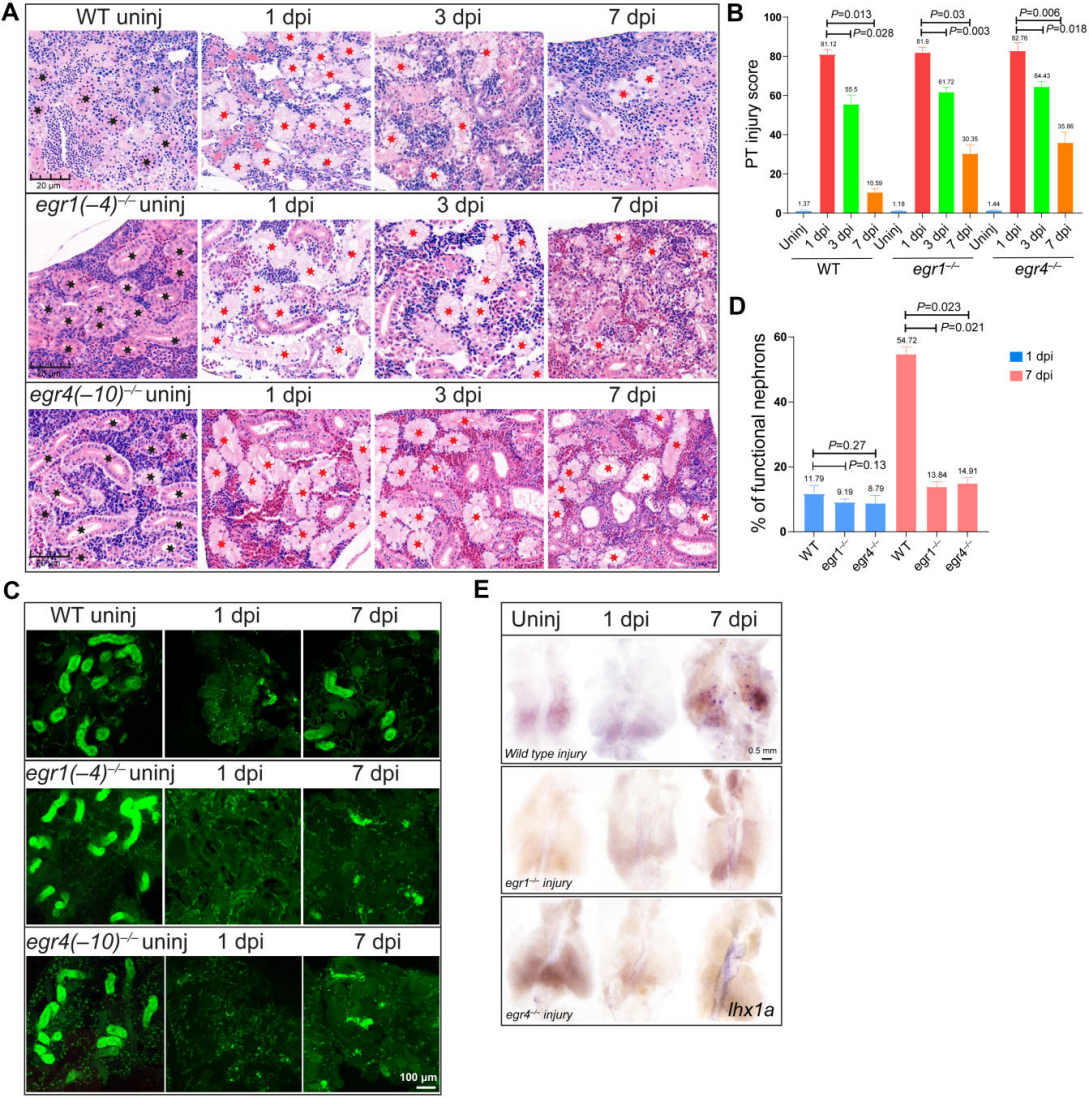
Loss of *egr1* or *egr4* inhibits renal regeneration. (**A** and **B**) HE staining and PT injury scoring of kidneys at the indicated time points (uninjured, 1 dpi, 3 dpi, and 7 dpi) in wild type, *egr1*^–/–^, and *egr4*^–/–^ zebrafish. (**A**) Tubules were severely damaged, with the tubule lumen greatly dilated at 1 dpi. In wild type kidneys, structures partially recovered at 3 dpi and most tubules were restored at 7 dpi. However, the regeneration process was delayed in mutant kidneys after injury. Black stars indicate the normal tubules; red stars indicate the dilated tubules. (**B**) PT injury score was used to show the percentage of abnormal PTs (*n* = 4 or 5 different regions of each group). (**C** and **D**) Dextran-FITC labeling and quantification of kidneys at the indicated time points (uninjured, 1 dpi, and 7 dpi) in wild type, *egr1*^–/–^, and *egr4*^–/–^ zebrafish. (**C**) The dextran fluorescence signals indicating functional PTs in uninjured kidneys were lost at 1 dpi in all zebrafish lines. Nephron PT function was partially restored in the wild type kidney at 7 dpi, but fewer and smaller PT signals were found in mutant lines. (**D**) The percentage of functional nephron PTs (*n* = 4 or 5 different regions of each group). (**E**) WISH images of *lhx1a* in control, *egr1*^–/–^, and *egr4*^–/–^ zebrafish at the indicated time points (uninjured, 1 dpi, and 7 dpi). Dot-like signals were significant in wild type kidneys at 7 dpi but hardly detected in uninjured kidneys, wild type kidneys at 1 dpi, or mutant injured kidneys.

Kidneys were collected at different time points (uninjured, 1 dpi, 3 dpi, 7 dpi, and 15 dpi) for morphological and functional examination. Hematoxylin–eosin (HE) staining revealed that renal tubules were severely damaged at 1 dpi in wild type, *egr1*^–/–^, and *egr4*^–/–^ kidneys (Figure 3A and B; Supplementary Figure S3A and B). When tubule regeneration was progressing from 3 dpi, the area of damaged renal tubules was much larger in *egr1*^–/–^ and *egr4*^–/–^ kidneys than in the wild type group (Figure 3A and B). In particular, at 15 dpi, the renal tubules completed structural regeneration in wild type kidneys, while there were still dilated tubules in *egr1*^–/–^ and *egr4*^–/–^ kidneys, indicating that *egr1* and *egr4* knockout delayed renal regeneration after 3 dpi (Supplementary Figure S3A and B). Kim1 immunofluorescence staining also confirmed the aggravation of kidney injury in *egr1*^–/–^ and *egr4*^–/–^ mutants at 3 dpi (Supplementary Figure S3C). Nephron function was subsequently evaluated by intraperitoneal injection of Dextran-FITC, which is absorbed by PTs and labels functional tubules (Wingert et al., 2014). As revealed by Dextran fluorescence, most nephrons lost their function at 1 dpi in wild type (a small number of nephrons still functioning), *egr1*^–/–^, and *egr4*^–/–^ kidneys (Figure 3C and D). Dextran fluorescence reappeared at 7 dpi in wild type kidneys, implying that nephrons began to regain the physiological function, while small and malformed signals were found in *egr1*^–/–^ and *egr4*^–/–^ kidneys, revealing a defect in renal regeneration (Figure 3C and D).

*lhx1a* is a marker gene of nephron progenitors (Diep et al., 2011). To determine the distribution of progenitors and the status of kidney regeneration, WISH was performed with the *lhx1a* probe. Several dot-like signals were present in wild type kidneys at 7 dpi, while such signals were hardly found in *egr1*^–/–^ and *egr4*^–/–^ kidneys from 1 dpi to 7 dpi (Figure 3E), suggesting the decreased renal progenitors in *egr1*^–/–^ and *egr4*^–/–^ injured kidneys. We concluded that *egr1* and *egr4* promote kidney regeneration through the regulation of renal progenitors after gentamicin-indcued AKI.

### Cell apoptosis and proliferation are disrupted in egr1 and egr4 mutants

**Figure 4:**
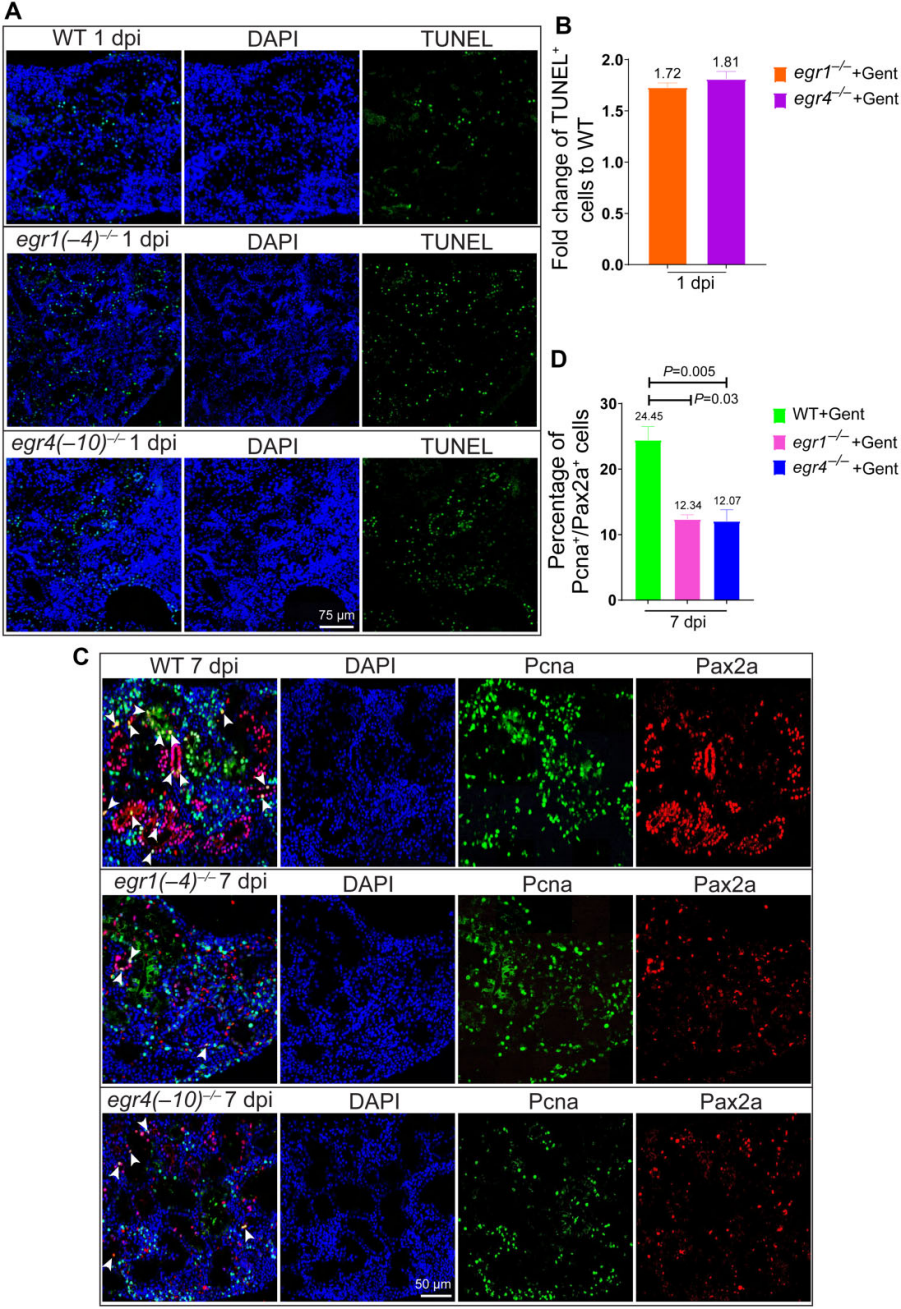
Cell apoptosis and proliferation are disrupted in *erg1*^–/–^ or *egr4*^–/–^ injured kidney. (**A** and **B**) TUNEL assay of kidneys at 1 dpi in wild type, *egr1*^–/–^, and *egr4*^–/–^ zebrafish. TUNEL^+^ cells were much more in mutants than in the wild type after kidney injury. TUNEL was repeated three times, and representative data are shown. (**C** and **D**) Pax2a and Pcna double immunofluorescence staining of kidneys from control, *egr1*^–/–^, and *egr4*^–/–^ zebrafish at 7 dpi. Pax2a^+^, Pcna^+^, and Pax2a^+^/Pcna^+^ cells were much less in mutants than in the wild type kidney. White arrowheads indicate the double-labeling cells. The percentage of Pax2a^+^/Pcna^+^ cells was quantified (*n* = 4 or 5 different regions of each group). Immunofluorescence was repeated three times and representative data are shown. Gent, gentamicin.

TUNEL assay was performed to detect tubular cell death. At 1 dpi, TUNEL^+^ cells in *egr1*^–/–^ and *egr4*^–/–^ kidneys were 1.72 and 1.81 times more than that in the wild type kidney, respectively (Figure 4A and B). In addition, cell apoptosis was barely detected in uninjured kidneys (Supplementary Figure S4A).

Pcna and Pax2a were chosen to label dividing cells and dedifferentiated PT cells, respectively. Immunofluorescence staining showed that at 7 dpi, both Pcna^+^ cells and Pax2a^+^ cells were much less in *egr1*^–/–^ and *egr4*^–/–^ kidneys than in the wild type kidney (Figure 4C). However, no difference in Pax2a or Pcna signals was found in uninjured kidneys (Supplementary Figure S4B). Moreover, as quantified by using ImageJ software, the proportions of Pcna^+^/Pax2a^+^ cells in *egr1*^–/–^ and *egr4*^–/–^ kidneys (12.34% and 12.07%, respectively) were significantly lower than that in the wild type (24.45%) at 7 dpi (Figure 4D), suggesting that the loss of *egr1* or *egr4* reduces renal tubular cell proliferation. Collectively, we propose that *egr1* and *erg4* promote renal regeneration by regulating cell proliferation and apoptosis in the kidney.

### foxm1 is upregulated by egr1 and egr4 to promote kidney regeneration

**Figure 5:**
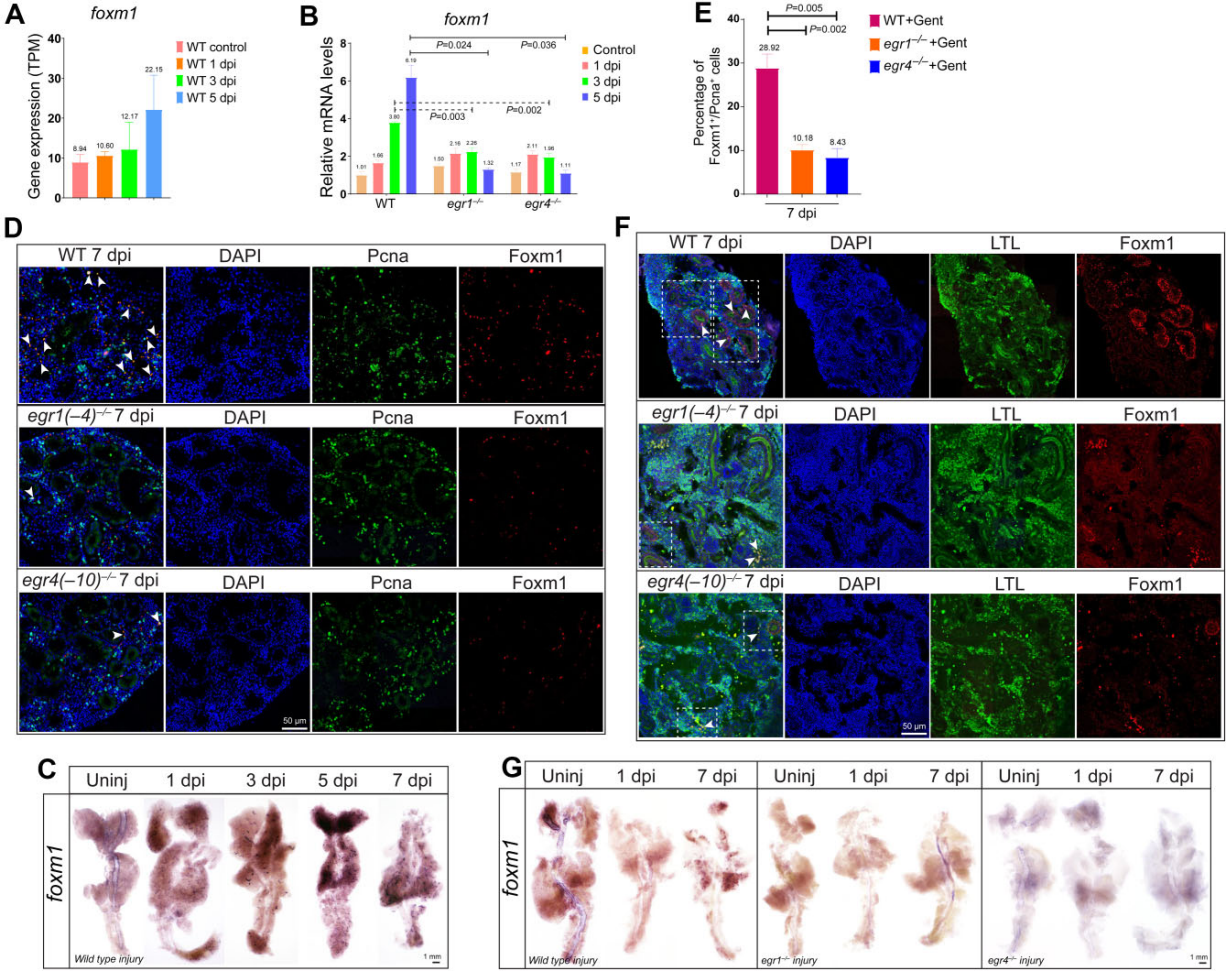
*foxm1* is the downstream target of *egr1* and *egr4*. (**A**) RNA-seq revealing that *foxm1* was gradually upregulated from 1 dpi to 5 dpi and peaked at 5 dpi. (**B**) qPCR analysis showing that *foxm1* was upregulated in wild type kidneys from 1 dpi to 5 dpi but the upregulation was disrupted in *egr1*^–/–^ and *egr4*^–/–^ kidneys. (**C**) WISH images of *foxm1* in wild type kidneys at the indicated time points (uninjured, 1 dpi, 3 dpi, 5 dpi, and 7 dpi). *foxm1* was expressed from 3 dpi to 7 dpi, with the expression peaking at 5 dpi and then decreasing. (**D** and **E**) Foxm1 and Pcna double immunofluorescence staining of wild type, *egr1*^–/–^, and *egr4*^–/–^ zebrafish kidneys at 7 dpi. Signals of Foxm1^+^, Pcna^+^, and Foxm1^+^/Pcna^+^ cells were all less in mutant kidneys than in the wild kidney. White arrowheads indicate Foxm1^+^/Pcna^+^ cells. The percentage of Foxm1^+^/Pcna^+^ cells was quantified (*n* = 4 or 5 different regions of each group). Immunofluorescence was repeated three times, and representative data are shown. (**F**) Foxm1 immunofluorescence with LTL staining at 7 dpi. Foxm1 was mostly expressed in LTL-labeled PTs, distributed in the nucleus and cytoplasm, and the expression decreased in mutant kidneys. White arrowheads indicate the cells with Foxm1 signals merged with DAPI; dashed lines represent the expression of Foxm1 in tubules. (**G**) WISH images of *foxm1* in wild type, *egr1*^–/–^, and *egr4*^–/–^ zebrafish kidneys at the indicated time points (uninjured, 1 dpi, and 7 dpi). *foxm1* was expressed in wild type injured kidneys at 7 dpi but hardly detected in uninjured kidneys, wild type kidneys at 1 dpi, or mutant injured kidneys.

Among the DEGs determined by RNA-seq of wild type regenerating kidneys, *foxm1* was identified probably regulated by *egr1* and *egr4*. It has been reported that *foxm1* is involved in kidney repair by driving tubular cell proliferation (Chang-Panesso et al., 2019), and its expression gradually increases during renal regeneration. Indeed, *foxm1* was upregulated in wild type kidneys from 1 dpi to 5 dpi (Figure 5A; Supplementary File 3). qPCR analysis indicated that *foxm1* levels significantly increased in wild type regenerating kidneys from 1 dpi to 5 dpi but did not change in *egr1*^–/–^ and *egr4*^–/–^ regenerating kidneys (Figure 5B). The comparison between the expression patterns of *foxm1* and *egr1*/*egr4* revealed that *egr1, egr4*, and *foxm1* were all upregulated after injury, with *egr1* and *egr4* peaking at 1 dpi, *foxm1* peaking at 5 dpi, and *egr1/egr4* levels ∼4/22-fold higher than the *foxm1* level (Figures 1A, B and 5A, B), suggesting that *egr1* and *egr4* might be the regulators of the *foxm1* gene.

Previous reports showed that *foxm1* is expressed in tubular epithelial cells in injured mouse and human kidneys (Chang-Panesso et al., 2019). Here, WISH results showed that *foxm1* was not detected in uninjured kidneys or wild type kidneys at 1 dpi, while its expression occurred in regenerating tubules at 3 dpi, peaked at 5 dpi, and then decreased (Figure 5C) confirming the upregulation of *foxm1* expression in tubular cells during renal regeneration.

Next, we assessed *foxm1* expression in *egr1*^–/–^ and *egr4*^–/–^ injured kidneys. Immunofluorescence staining detected few Foxm1^+^ cells in uninjured kidneys and there was no difference among *egr1*^–/–^, *egr4*^–/–^, and wild type groups (Supplementary Figure S5A). At 7 dpi, *foxm1* was clearly upregulated, but Foxm1^+^ cells were much less in *egr1*^–/–^ and *egr4*^–/–^ kidneys than in the wild type kidney (Figure 5D). In addition, the proportions of Foxm1^+^/Pcna^+^ cells in *egr1*^–/–^ and *egr4*^–/–^ kidneys (10.18% and 8.43%, respectively) were significantly lower than that in wild type regenerating kidney (28.92%) at 7 dpi (Figure 5E).

To confirm the exact location of *foxm1* in tubular cells, we performed co-staining for Foxm1 and Lotus tetragonolobus lectin (LTL), a marker of PTs (Cachat et al., 2003; Bonegio et al., 2011; McCampbell et al., 2015). Foxm1^+^ cells were hardly present in uninjured PTs (Supplementary Figure S5B). At 7 dpi, Foxm1 fluorescence was merged with LTL fluorescence that was distinct in PTs (Figure 5F). Notably, Foxm1 was located in both the nucleus and cytoplasm, as revealed by DAPI staining (Figure 5F). Finally, WISH confirmed that *foxm1* was expressed in wild type kidneys at 7 dpi, but not in uninjured kidneys, wild type kidneys at 1 dpi, or *egr1*^–/–^ and *egr4*^–/–^ kidneys at 1 dpi and 7 dpi (Figure 5G).

These results provide strong evidence that *foxm1* is expressed in renal tubular cells and regulated by *erg1* and *egr4* in zebrafish regenerating kidney.

### foxm1 knockout impairs renal regeneration

**Figure 6:**
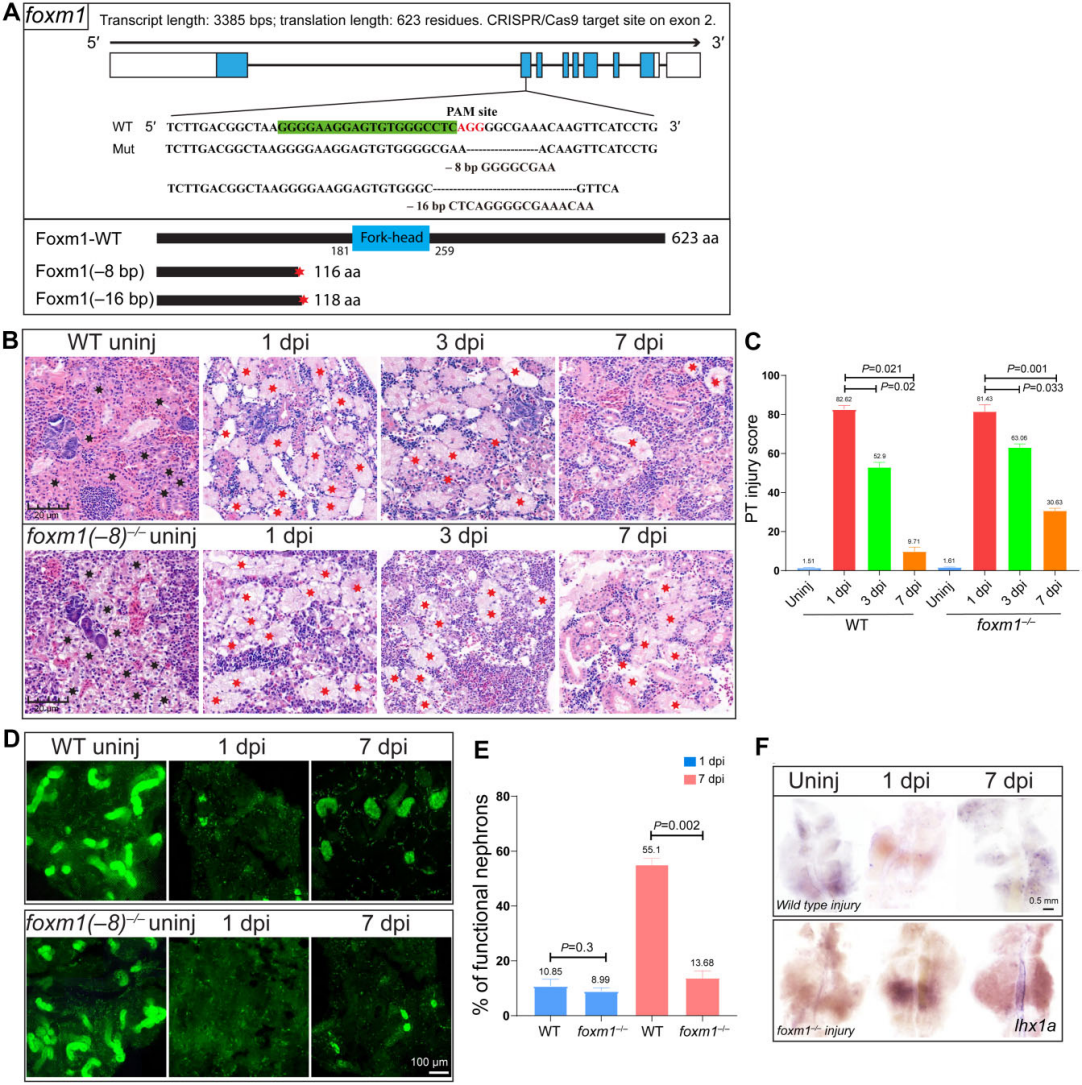
*foxm1* deficiency inhibits tubule regeneration. (**A**) Upper: the Cas9 target site of *foxm1* on exon 2 and the obtained –8 bp and –16 bp mutant alleles. Lower: full-length and truncated Foxm1 proteins of 623 aa, 116 aa, and 118 aa, respectively. (**B** and **C**) HE staining and PT injury scoring of kidneys at the indicated time points (uninjured, 1 dpi, 3 dpi, and 7 dpi) in wild type and *foxm1*^–/–^ zebrafish. (**B**) Tubules were severely damaged and the tubule lumen was dilated at 1 dpi. In wild type kidneys, structures partially recovered at 3 dpi and most tubules were restored at 7 dpi. However, regeneration was delayed in mutant injured kidneys. Black stars indicate the normal tubules, and red stars indicate damaged tubules. (**C**) The percentage of abnormal PTs was quantified as the PT injury score (*n* = 4 or 5 different regions of each group). (**D** and **E**) Representative dextran fluorescence signals and percentage of functional nephrons (*n* = 4 or 5 different regions of each group) in wild type and *foxm1*^–/–^ zebrafish kidneys at the indicated time points (uninjured, 1 dpi, and 7 dpi). (**F**) WISH images of *lhx1a* showing significant dot-like signals in wild type kidneys at 7 dpi, but hardly detected in uninjured kidneys, wild type kidneys at 1 dpi, or mutant injured kidneys.

To further study the role of *foxm1* in renal regeneration after AKI, two mutant alleles for *foxm1* were maintained, i.e. –8 bp and –16 bp, which translated truncated proteins at 116 aa and 118 aa, respectively (Figure 6A; Supplementary Figure S6A). Homozygous mutant offsprings survived and showed a normal phenotype, and thus were used for subsequent experiments. There was no difference in Foxm1 fluorescence between *foxm1*^–/–^ and wild type uninjured kidneys, as little fluorescence was found in both groups, while the Foxm1 fluorescence in *foxm1*^–/–^ regenerating kidney was much weaker than that in the wild type kidney at 7 dpi (Supplementary Figure S6B). The proportion of Foxm1^+^/Pcna^+^ cells sharply decreased from 28.92% in the wild type kidney to 3.42% in the *foxm1*^–/–^ kidney at 7 dpi (Supplementary Figure S6C). WISH also showed that *foxm1* was only expressed in the wild type kidney at 7 dpi but not expressed in *foxm1*^–/–^ kidneys at 1 dpi and 7 dpi (Supplementary Figure S6D). Therefore, *foxm1* was successfully knocked out, and loss of *foxm1* inhibited cell proliferation during renal regeneration.

HE staining revealed severe tubule injury in both *foxm1*^–/–^ and wild type kidneys at 1 dpi, but the area of injured tubules was much larger in *foxm1*^–/–^ kidneys than in wild type kidneys at 3 dpi and 7 dpi (Figure 6B and C), suggesting impaired kidney regeneration after AKI in the *foxm1*^–/–^ mutant. Kim1 immunofluorescence also showed more injured tubules in the *foxm1*^–/–^ kidney at 3 dpi (Supplementary Figure S6E). At 7 dpi, the number of Dextran-labeled tubules in *foxm1*^–/–^ regenerating kidney was much lower than that in the wild type kidney, and the size of the labeled tubules was smaller (Figure 6D and E). Furthermore, *lhx1a* expression was present in wild type kidneys at 7 dpi but barely detected in *foxm1*^–/–^ injured kidneys (Figure 6F). These results clearly indicated that the loss of *foxm1* expression reduced *lhx1a* expression in injured kidney and inhibited renal regeneration.

### foxm1 inhibits cell apoptosis and promotes proliferation during renal regeneration

**Figure 7:**
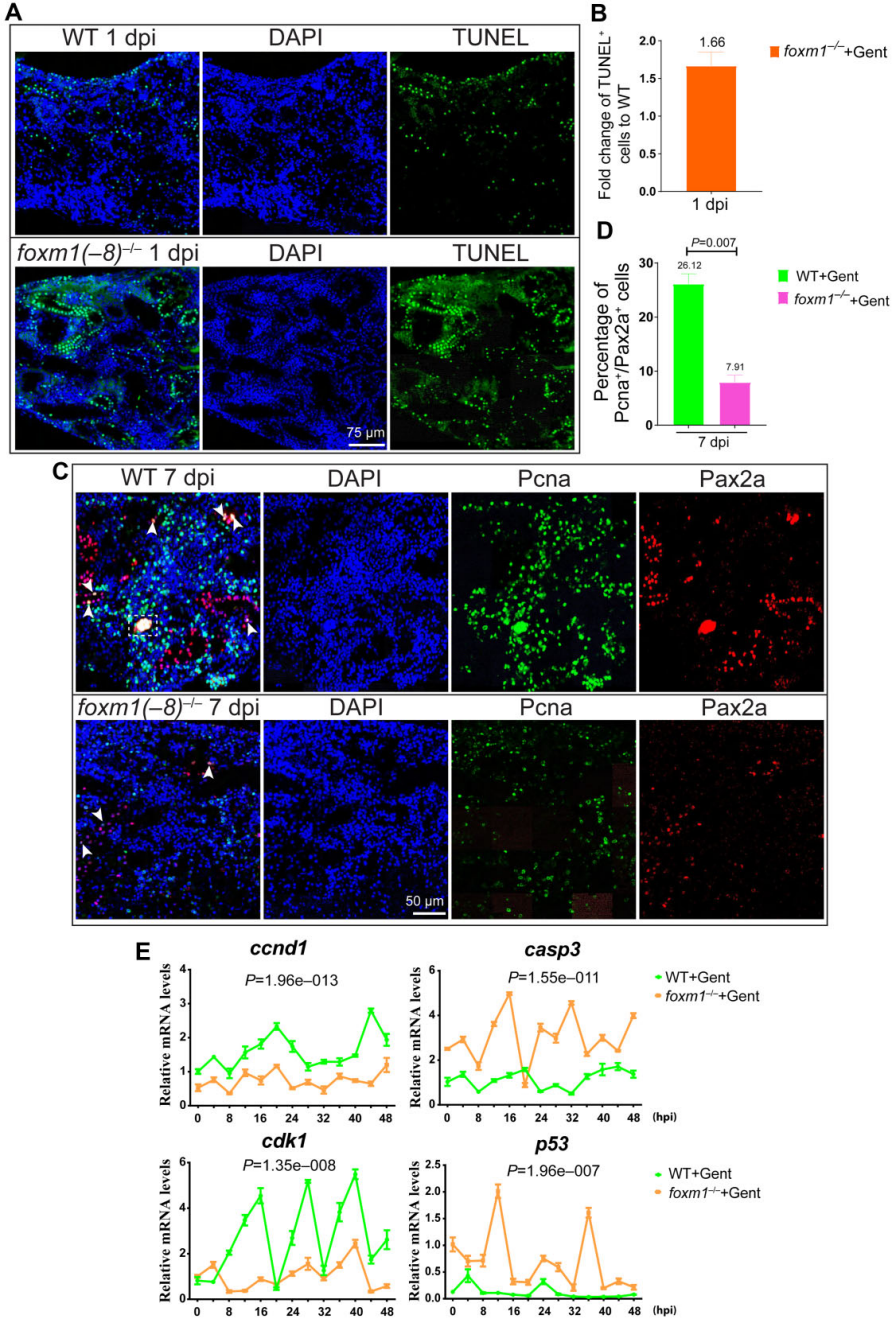
Loss of *foxm1* induces apoptosis and inhibits proliferation of tubular cells. (**A** and **B**) TUNEL assay and quantification showing much more TUNEL^+^ cells in the *foxm1*^–/–^ kidney than in the wild type kidney after injury. TUNEL was repeated three times, and representative data are shown. (**C** and **D**) Pax2a and Pcna double immunofluorescence staining indicating much less Pax2a^+^ and Pcna^+^ cells in the *foxm1*^–/–^ kidney than in the wild type kidney at 7 dpi. The percentage of Pax2a^+^/Pcna^+^ cells also dramatically decreased in the *foxm1*^–/–^ kidney. Arrowheads indicate Pax2a^+^/Pcna^+^ cells, and dashed lines represent renal progenitor cell aggregation. Immunofluorescence was repeated three times, and representative data are shown. (**E**) qPCR analysis of proliferation and apoptosis-related gene expression in wild type and *foxm1*^–/–^ within 48 hpi of gentamicin treatment. The expression of cell proliferation genes *ccnd1* and *cdk1* was downregulated, while the expression of the apoptosis marker genes *casp3* and *p53* was upregulated in *foxm1*^–/–^ injured kidneys.

Previous studies demonstrated that *foxm1* promotes tubular cell proliferation (Chang-Panesso et al., 2019; Sinha et al., 2020) and inhibits cell apoptosis in cancer (Zhou et al., 2021). Here, TUNEL^+^ cells in the *foxm1*^–/–^ kidney were 1.66 times more than that in the wild type kidney at 1 dpi (Figure 7A and B), but there was no difference between wild type and *foxm1*^–/–^ uninjured kidneys (Supplementary Figure S7A), indicating that loss of *foxm1* promotes renal tubular cell apoptosis after AKI. Similarly, while no difference in Pax2a or Pcna fluorescence was found in uninjured kidneys (Supplementary Figure S7B), both Pcna^+^ and Pax2a^+^ cells were much less in the *foxm1*^–/–^ kidney than in the wild type kidney at 7 dpi, and the percentage of Pcna^+^/Pax2a^+^ cells dropped from 26.12% in the wild type kidney to 7.91% in the *foxm1*^–/–^ kidney (Figure 7C and D) indicating that loss of *foxm1* expression inhibits tubular cell proliferation in regenerating kidney. Furthermore, qPCR analysis of mRNA levels of *ccnd1, cdk1, p53*, and *casp3*, marker genes of cell proliferation and apoptosis (Zou et al., 2014; Yu et al., 2016), within 48 h post-injury (hpi) showed that the cell proliferation-associated genes *ccnd1* and *cdk1* were dramatically downregulated in *foxm1*^–/–^ injured kidneys, while the cell apoptosis-associated genes *p53* and *casp3* were upregulated in *foxm1*^–/–^ injured kidneys (Figure 7E), confirming that *foxm1* regulates renal tubular cell proliferation and apoptosis-related gene expression. Overall, *foxm1* is involved in keeping the balance between renal tubular cell proliferation and apoptosis during regeneration.

### Egr1 and Egr4 directly bind to foxm1 promoter to regulate gene expression

**Figure 8:**
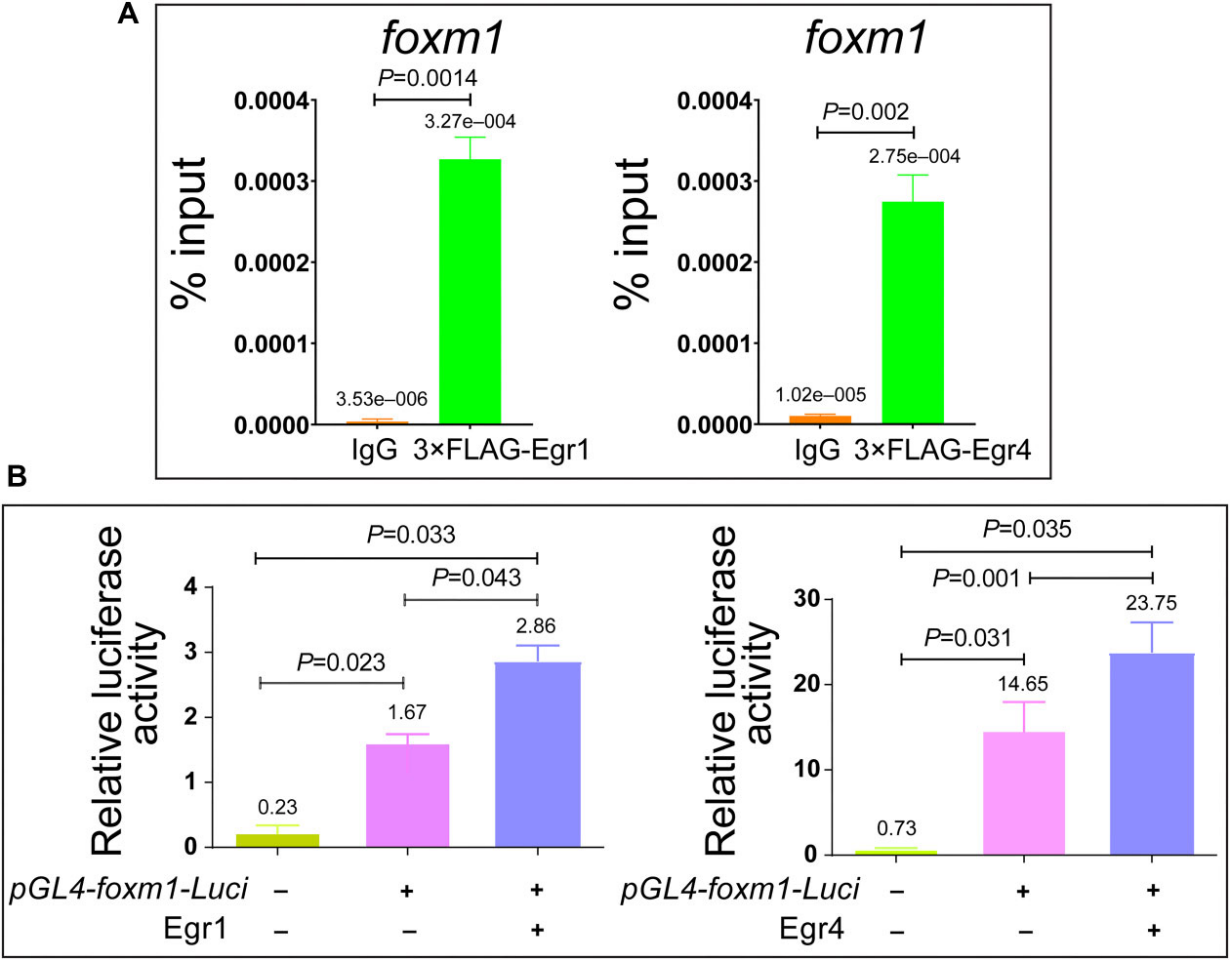
*egr1* and *egr4* regulate *foxm1* expression by directly binding to its promoter region. (**A**) ChIP–PCR assay indicating the enrichment of Egr1 and Egr4 in *foxm1* promoter. ChIP experiments were repeated three times. (**B**) Luciferase assay showing the enhancement of *foxm1* promoter activity by Egr1 or Egr4 expression. Luciferase experiments were repeated three times.

To examine whether the transcription factors Egr1 and Egr4 bind to *foxm1* promoter directly to regulate gene expression, the mRNA encoding 3×FLAG-tagged Egr1 or Egr4 was injected into 1-cell stage zebrafish embryos. Chromatin immunoprecipitation (ChIP) performed using FLAG antibodies revealed that 3×FLAG-Egr1 and 3×FLAG-Egr4 bound to *foxm1* promoter, since the ChIP enrichment efficiency of Egr1 or Egr4 was much higher in Egr1- or Egr4-overexpressing embryos than in the negative control group (Figure 8A).

Next, *pGL4-foxm1-Luciferase* reporter plasmid was constructed with a 2926-bp promoter sequence and transfected with or without Egr1- or Egr4-expressing plasmid into HEK293T cells. Since the luciferase activity gradually increased with the increasing concentration of *egr1* or *egr4* plasmid ≤1 μg but decreased when the concentration of plasmid was >1 μg (Supplementary Figure S8A), 500 ng *egr1* or *egr4* plasmids were transfected into cells. The results showed that Egr1 or Egr4 increased the luciferase activities (Figure 8B), suggesting that Egr1 and Egr4 directly bind to and activate *foxm1*.

## Discussion

It is widely accepted that during renal regeneration following AKI, the surviving epithelial cells that originally reside in the normal kidney are the source of regeneration (Duffield and Humphreys, 2011; Chang-Panesso et al., 2019), while extra-renal cells only contribute indirectly to tubular regeneration with potential paracrine factors (Genheimer et al., 2012; Johansson 2014; Zhang et al., 2024). Renal tubule epithelial cells have a strong ability to proliferate, dedifferentiate, and migrate, and they are primarily responsible for tubular regeneration after AKI (Chen et al., 2018; Li and Li, 2019). There is a debate on whether the surviving tubular cells, which contribute to regeneration, belong to tubular stem cells or progenitor-like cells that arise from epithelium dedifferentiation and possess progenitor cell phenotype and abilities. The generally accepted conclusion is that the injured tubular cells regain a reversible potential, undergoing dedifferentiation, proliferation, and differentiation to restore tubules (Johansson 2014; Chang-Panesso et al., 2019; Zhang et al., 2024). Here, we found that *egr* genes were significantly upregulated after kidney injury according to bulk RNA-seq data. In particular, *egr1* and *egr4* were rapidly and transiently upregulated after AKI, highlighting their role in kidney regeneration.

*egr1* can be a trigger and a core factor in cell growth, proliferation, differentiation, migration, and apoptosis in tissue repair and regeneration (Zins et al., 2014; Wang et al., 2021). A recent study indicated that *egr1* is involved in mouse kidney repair by regulating the expression of downstream genes to drive cell proliferation and migration of tubular epithelium (Chen et al., 2022). However, until now, the role of *egr4* in the regulation of tissue repair and regeneration has not been well studied.

Our findings revealed that *egr1* and *egr4* knockout exacerbated kidney injury and inhibited tubule regeneration, showing dramatically decreased expression of *lhx1a*, significantly reduced Pcna^+^/Pax2a^+^ and Pax2a^+^ cells, and increased TUNEL^+^ tubular cells, consistent with previous studies showing that *egr* genes have the ability to regulate cell proliferation and apoptosis as well as progenitors in the injured tissue (Zhou et al., 2022; Zou and Zeng, 2023).

Foxm1 is involved in tissue regeneration after injury through regulating downstream gene expression (Krupczak-Hollis et al., 2004; Cheng et al., 2016). *foxm1* is able to regulate cell proliferation and apoptosis (Zhou et al., 2021). A recent study reported that *foxm1* is upregulated and mostly localized in the S3 segment of the nephron, which is the area most susceptible to injury in murine and human kidneys, indicating that *foxm1* is an important regulator of PT cell proliferation after injury (Chang-Panesso et al., 2019). In our study, *foxm1* was also highly expressed in injured tubules, as revealed by WISH and immunofluorescence experiments. *foxm1* deficiency caused a decrease in the number of proliferating cells and progenitor-like cells during kidney regeneration and promoted tubular cell apoptosis after AKI. Our results also revealed that the proliferation-related genes (*ccnd1* and *cdk1*) were downregulated in *foxm1*^–/–^ mutants and the apoptotic genes (*p53* and *casp3*) were upregulated. These changes were responsible for impaired kidney regeneration in the *foxm1*^–/–^ kidney after injury.

*foxm1* is known as a regulator of cell proliferation and apoptosis in tissue repair and regeneration. However, which genes are upstream of *foxm1* and how these genes regulate *foxm1* expression are not yet clear. Here, *egr1* and *egr4* were found to upregulate *foxm1* expression by binding to its promoter region. This finding provides a new insight into the role and mechanism of *egr1*/*egr4* in promoting kidney regeneration and the relationship between *egr1*/*egr4* and *foxm1*.

In conclusion, (i) *egr1* and *egr4* can be quickly and transiently induced after AKI; (ii) *foxm1* promotes kidney regeneration by promoting tubular epithelial cell proliferation and inhibiting cell apoptosis; and (iii) *foxm1* is a novel downstream effector of *egr1* and *egr4*, promoting zebrafish renal regeneration after injury.

## Materials and methods

### Zebrafish maintenance

Fish strains were raised in a constant-temperature water circulation system (28.5°C) under a 14-h light/10-h dark cycle.

### Generation of mutant zebrafish lines by CRISPR/Cas9

To generate zebrafish mutants, guide RNAs were synthesized using the commercial MEGA Shortscript T7 kit (#AM1354200, Ambion), mixed with Cas9 protein (#A36498, Invitrogen), and injected into 1-cell stage embryos to knock out the target gene.

### Induction of AKI by gentamicin

AKI was induced by gentamicin injection (80 mg/kg body weight) in 5-month-old adult wild type and mutant zebrafish (average weight 0.5 g). Zebrafish injected with a similar volume of PBS served as a control.

### Bulk RNA-seq and heatmap analysis

For bulk RNA-seq experiments, at least two technical replicates with 8–10 pooled kidneys at each time point were used. The RNA library construction and sequencing were performed by the BGI Company, Shenzhen, China, using a BGI-500 system. Heatmap analysis was performed using TPM, and the plot was made using pheatmap.

### WISH

To detect the expression of *slc20a1a, trpm7, slc12a1, slc12a3, lhx1a, egr1, egr4*, and *foxm1*, RNA probes were purified using mini-Quick Spin RNA Columns (Roche) and stored at −80°C in deionized formamide. WISH was performed according to the standard protocol (Diep et al., 2011; Liu et al., 2023).

### qPCR analysis

To detect the expression of genes of interest, qPCR was performed on a Bio-Rad instrument, and the data were processed by GraphPad Prism 9. The primers are listed in Supplementary Table S7.

### Dextran-FITC fluorescence

Dextran-FITC (#R-FD-003, RuiXi Bio) was intraperitoneally injected into zebrafish to evaluate the absorptive function of nephrons. The detailed procedure for analyzing Dextran-labeled PTs was previously described (Wingert et al., 2014).

### TUNEL assay

Apoptosis was examined using the TUNEL Apoptosis Detection Kit (#40307ES20, YEASEN) according to the manufacturer's instructions. Images were captured by a Leica confocal microscope (TCS SP8 STED).

### Statistical analysis

Statistical analysis was carried out using GraphPad Prism 9.0 software (GraphPad Software Inc.). Student's *t*-test was used to compare two groups, while analysis of variance (ANOVA) was used to compare multiple groups. All the reported error bars indicate the standard deviation. *P*-values are indicated in the figures to show the statistical significance. *P* < 0.05 was considered statistically significant.

### Ethics statement

Animal experiments and treatments were performed according to the Guide for Animal Care and Use Committee of the Institute of Hydrobiology, Chinese Academy of Sciences (IHB, CAS, Protocol No. 2016-018). All the relevant ethical regulations for animal use were strictly followed according to the Animal Experimental Ethical Inspection by IHB, No. IHB2024-0901.

### Data sharing statement

The raw data of bulk RNA-seq for regenerating kidneys, which is reported in this article, have been deposited in the Genome Sequence Archive in the National Genomics Data Center, China National Center for Bioinformation/Beijing Institute of Genomics, Chinese Academy of Sciences (accession number CRA014881) and are publicly accessible at https://ngdc.cncb.ac.cn/gsa/search/getSearchByAccession.

## Supplementary Material

mjaf026_Supplemental_Files

## Notes

**Author contributions:** X.H. performed the experiments and wrote the manuscript. Y.S. designed the study and supervised the project. Both authors critically reviewed and approved the final manuscript.
