# Supplementary material for: egr1 and egr4 regulate zebrafish renal regeneration by promoting foxm1 expression
Source: J Mol Cell Biol. 2025 Aug 15;17(6):mjaf026. doi: 10.1093/jmcb/mjaf026 (PMC12859677; doi:10.1093/jmcb/mjaf026)
Supplement: mjaf026_Supplemental_Files [file mjaf026_supplemental_files.zip › Supplementary material.pdf]

Supplementary material

egr1 and *egr4* regulate zebrafish renal regeneration by promoting *foxm1* expression

Xian He and Yuhua Sun*

* Correspondence to: Yuhua Sun, E-mail: sunyh@ihb.ac.cn; Tel: +86-27-68780718

Supplementary Files (Excel)

Supplementary File 1

Supplementary Table S1 CRISPR/Cas9 target sequences of *egr1*, *egr4*, and *foxm1*.

Supplementary Table S2 Screening primers for *egr1*, *egr4*, and *foxm1* mutants.

Supplementary Table S3 Primers of genes for probe used for *in situ* hybridization. PCR was used to amplify probe DNA models, T7 promoter sequence was added ahead of the reverse primer.

Supplementary Table S4 *foxm1* ChIP-qPCR primers.

Supplementary Table S5 Primers of *foxm1* for promoter amplification.

Supplementary Table S6 Coding sequence amplification primers of *egr1* and *egr4*.

Supplementary Table S7 qRT-PCR primers for genes used for regeneration experiments.

Supplementary File 2 DEGs of wild type regenerating kidneys extracted from bulk RNA sequencing.

Supplementary File 3 Gene expression (TPM) in kidneys extracted from wild type RNA sequencing.

Supplementary Figures

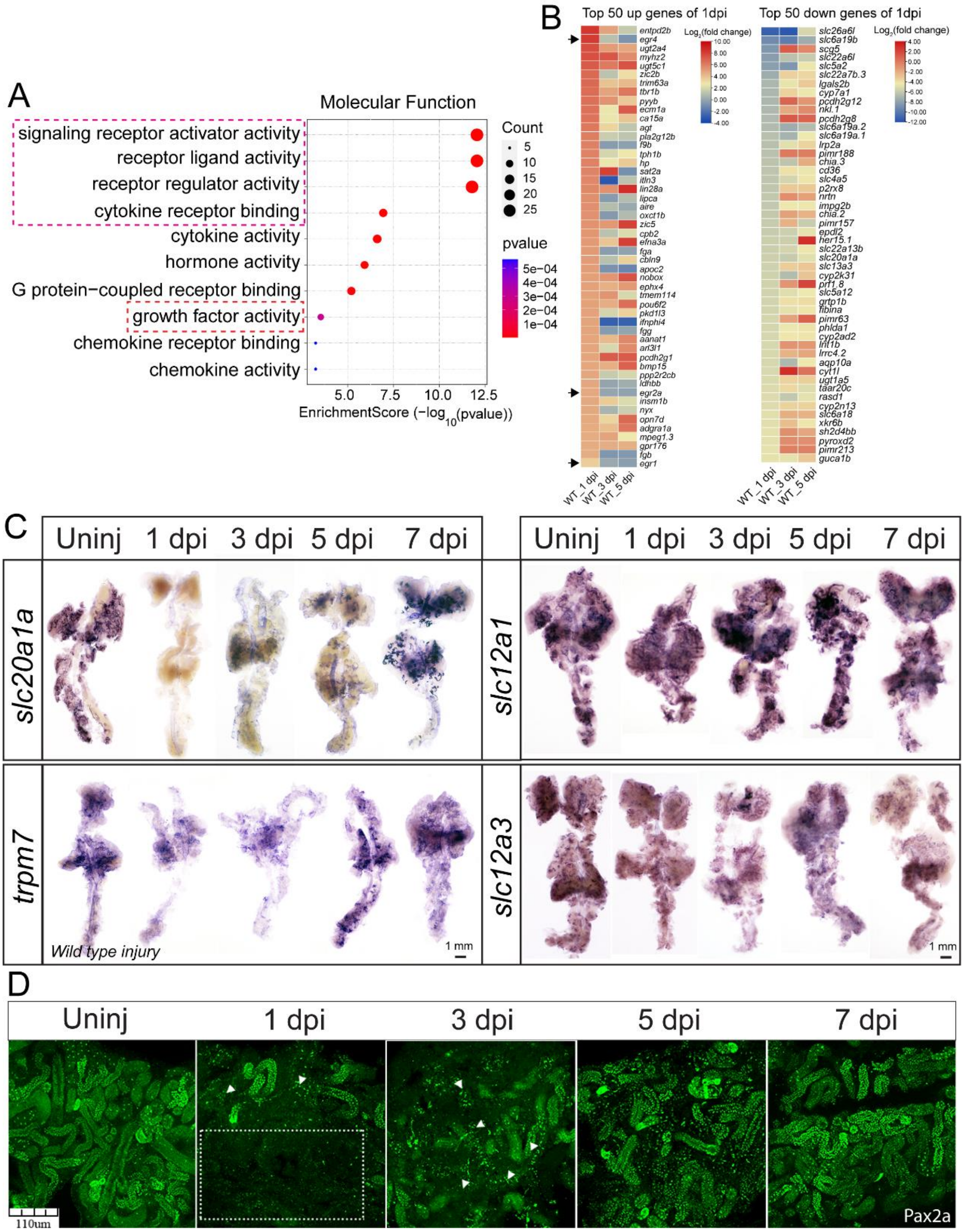

Supplementary Figure S1 Growth factors and ligand genes are enriched in injured tubules. (**A**) GO enrichment analysis of the upregulated DEGs at 1 dpi. Most genes involved in receptor activities are highlighted by the pink box, and the GO term of the growth factor activity is labeled by the red box. (**B**) Heatmap of the 50 top upregulated and downregulated DEGs from bulk RNA-sequencing. *egr4* expression was ranked as second at 1dpi; *egr1* was also dramatically expressed at 1dpi. (**C**) WISH assays of *slc20a1a*, *trpm7*, *slc12a1*, and *slc12a3* at the indicated time points (uninjured, 1 dpi, 3 dpi, 5 dpi, and 7 dpi). Results showing that *slc20a1a* and *trpm7* had the same

expression pattern hardly detected at 1 dpi while gradually recovered after 3 dpi. No difference was observed between *slc12a1* and *slc12a3* expression from 1 dpi to 7 dpi compared to uninjured kidneys. (**D**) Pax2a staining images from wild type kidneys at the indicated time points (uninjured, 1 dpi, 3 dpi, 5 dpi, and 7 dpi). Pax2a labeling tubule cells disappeared mostly at 1 dpi, and the dispersed tubule cells found at 3 dpi were arranged in a line. The labeled cells increased fast and spread along the tubules when regeneration occurred at 5–7 dpi. White arrow heads indicate the dispersed tubule cells, and the dashed line represents the areas that lost the tubules.

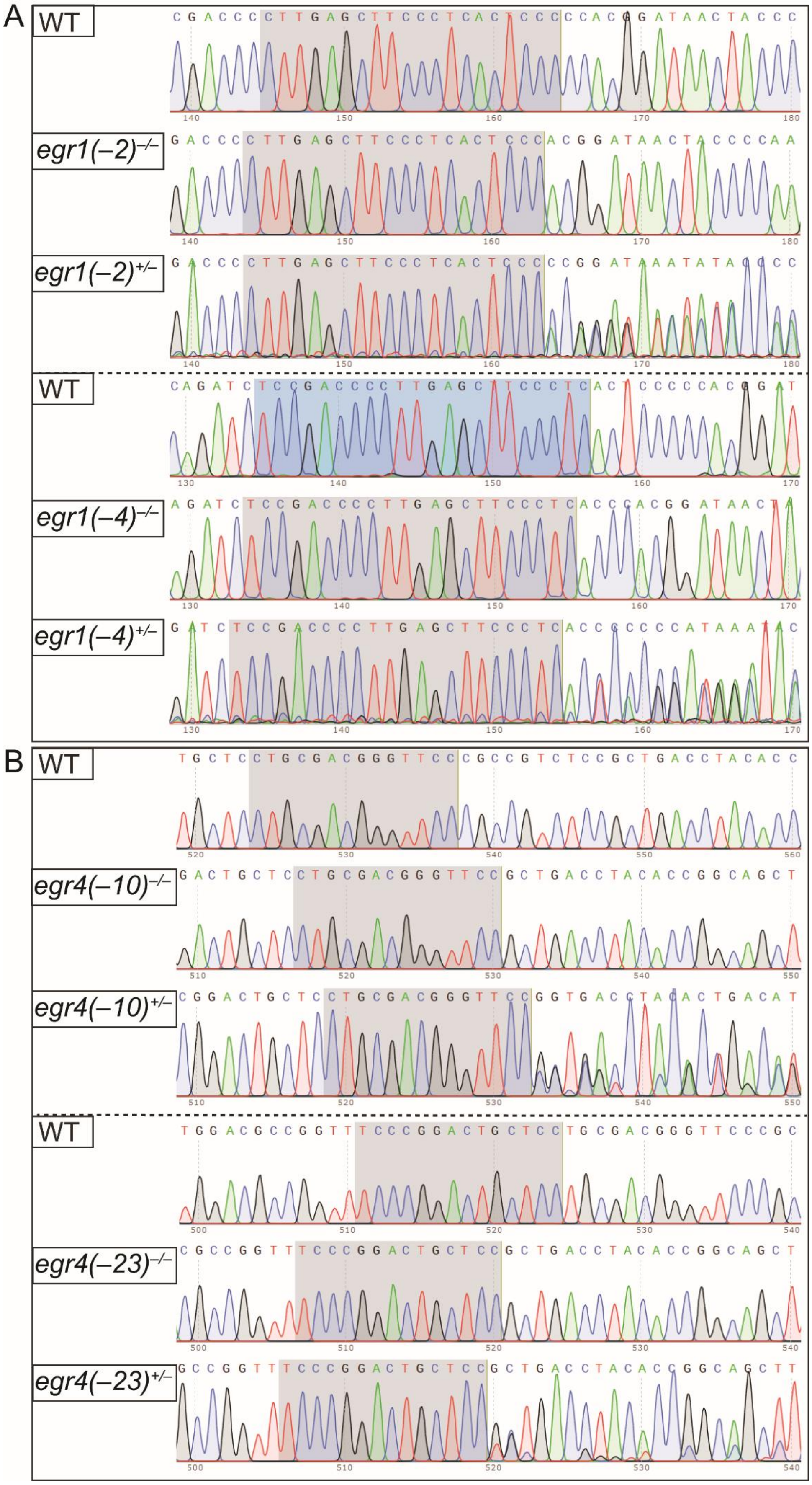

Supplementary Figure S2 Sequencing map of *egr1* and *egr4* mutants. (**A**) Maps for *egr1* mutant alleles. (**B**) Maps for *egr4* mutant alleles.

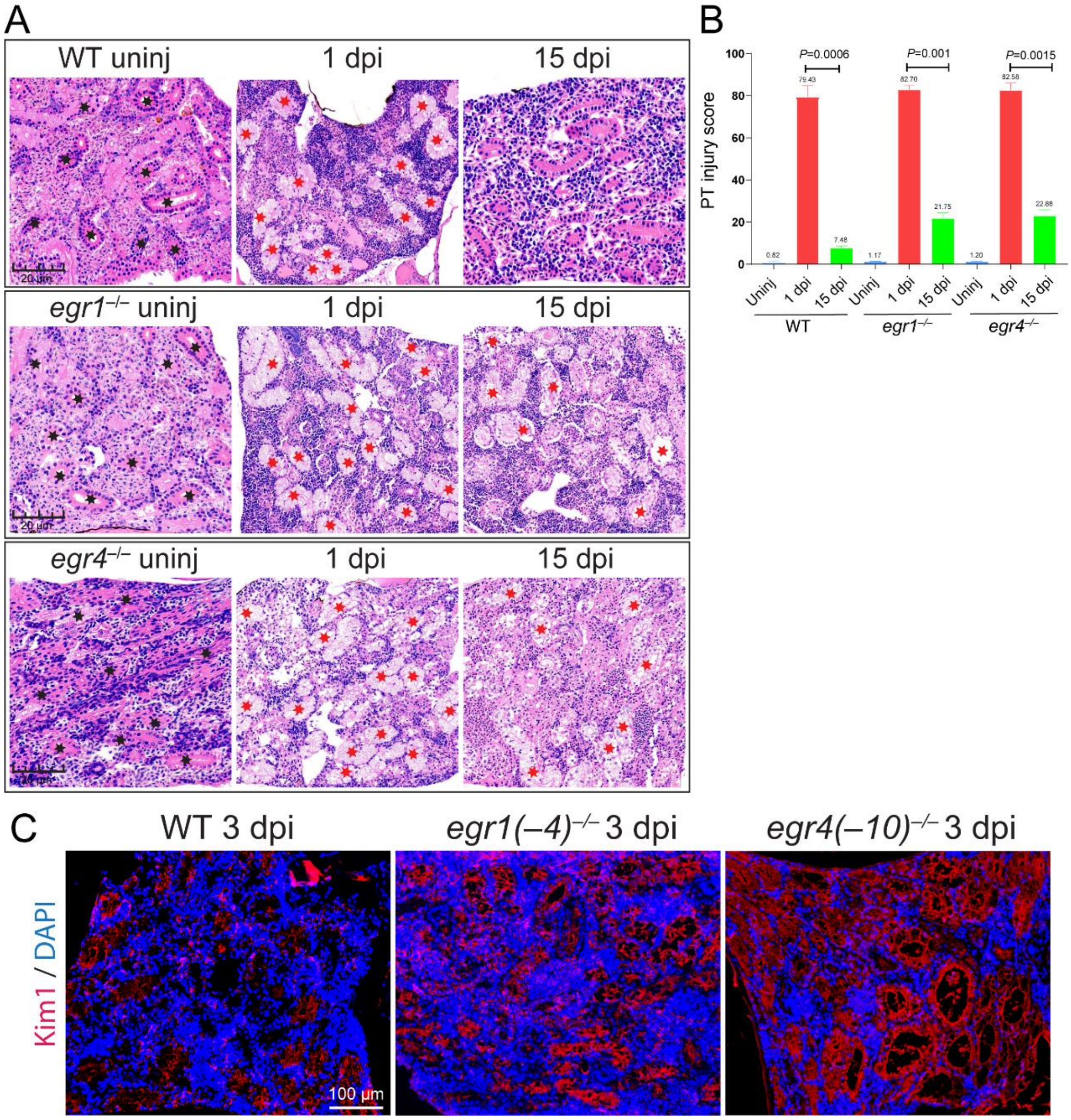

Supplementary Figure S3 *egr1* and *egr4* knockout exacerbates injury effect. (**A**) HE staining in *egr1*$^{-/-}$ and *egr4*$^{-/-}$ injured kidneys at 1 and 15 dpi. (**B**) Quantification of panel A (n = 4-5 different regions of each group) with p values. Gent: gentamicin. HE was repeated three times and representative data are shown. P values for the panel are indicated to show the statistical significance. (**C**) Kim1 IF of *egr1*$^{-/-}$ and *egr4*$^{-/-}$ kidneys at 3 dpi after AKI.

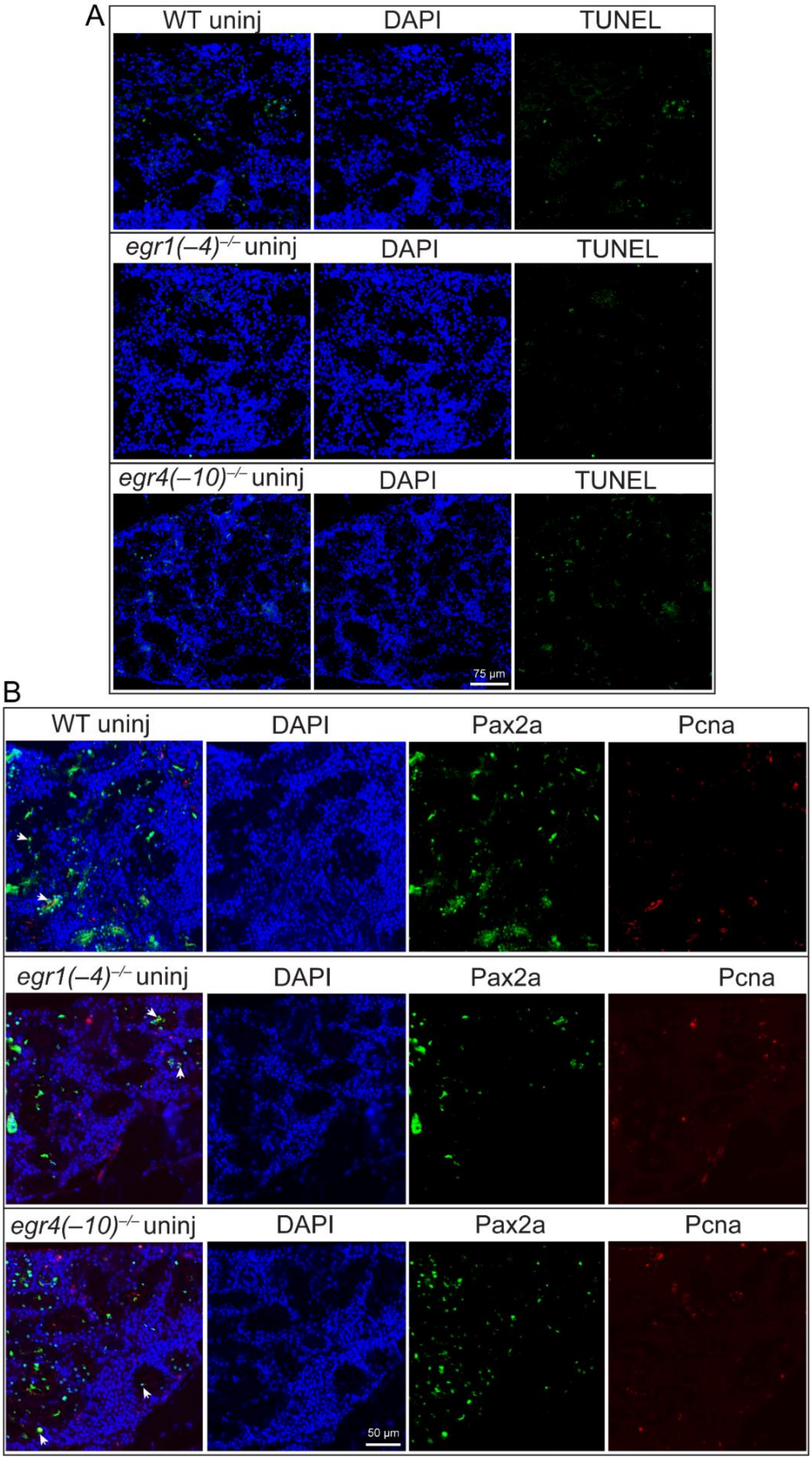

Supplementary Figure S4 No difference in cell apoptosis and proliferation in uninjured mutant kidneys. (**A**) TUNEL assay of wild type, *egr1*$^{-/-}$ and *egr4*$^{-/-}$ zebrafish uninjured kidneys. (**B**) Images of Pax2a and Pcna double IF from wild type, *egr1*$^{-/-}$ and *egr4*$^{-/-}$ uninjured kidneys. White arrow heads indicate merged staining cells.

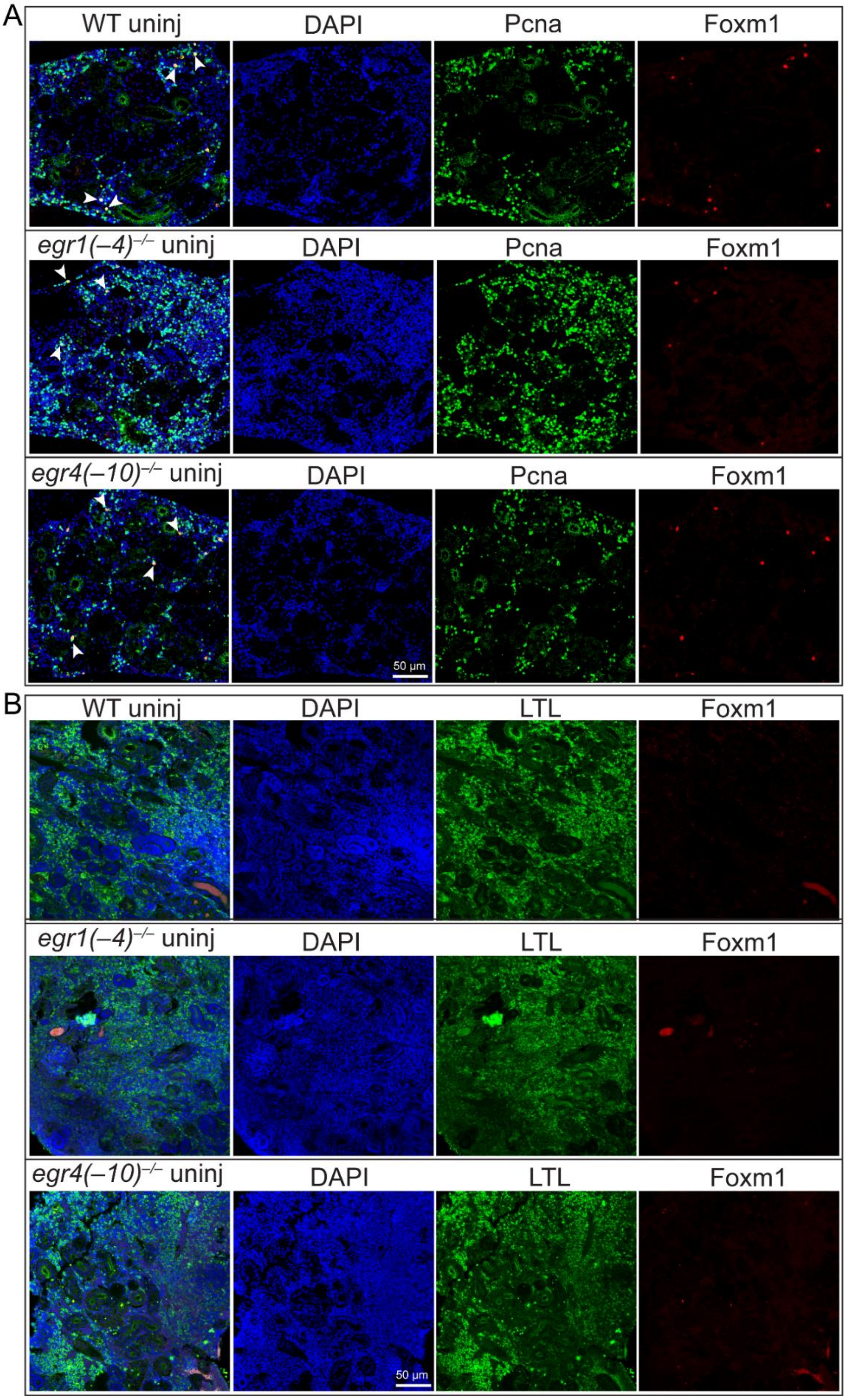

Supplementary Figure S5 *foxm1* is hardly detected in uninjured kidney. (**A**) Foxm1 and Pcna images from control, *egr1*$^{-/-}$, and *egr4*$^{-/-}$ uninjured kidneys. Arrow heads indicate the Foxm1^{+}/Pcna^{+} cells. (**B**) Foxm1 IF with LTL staining in uninjured kidneys from wild type, *egr1*$^{-/-}$, and *egr4*$^{-/-}$ zebrafish.

A

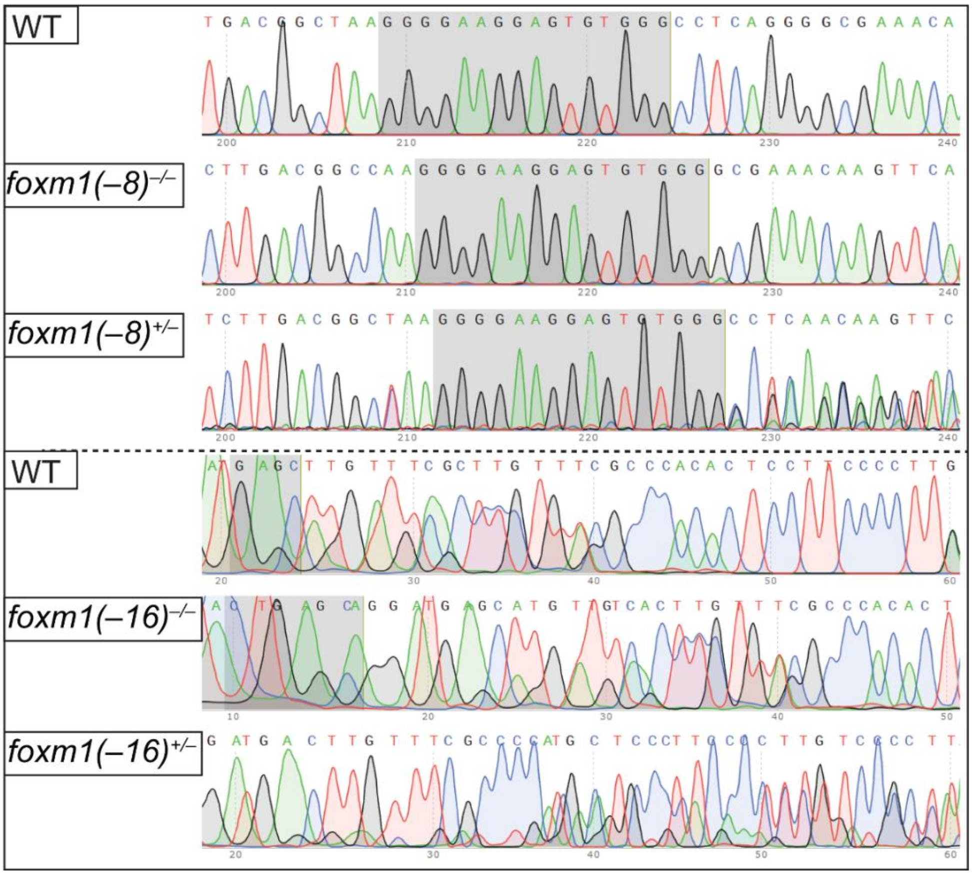

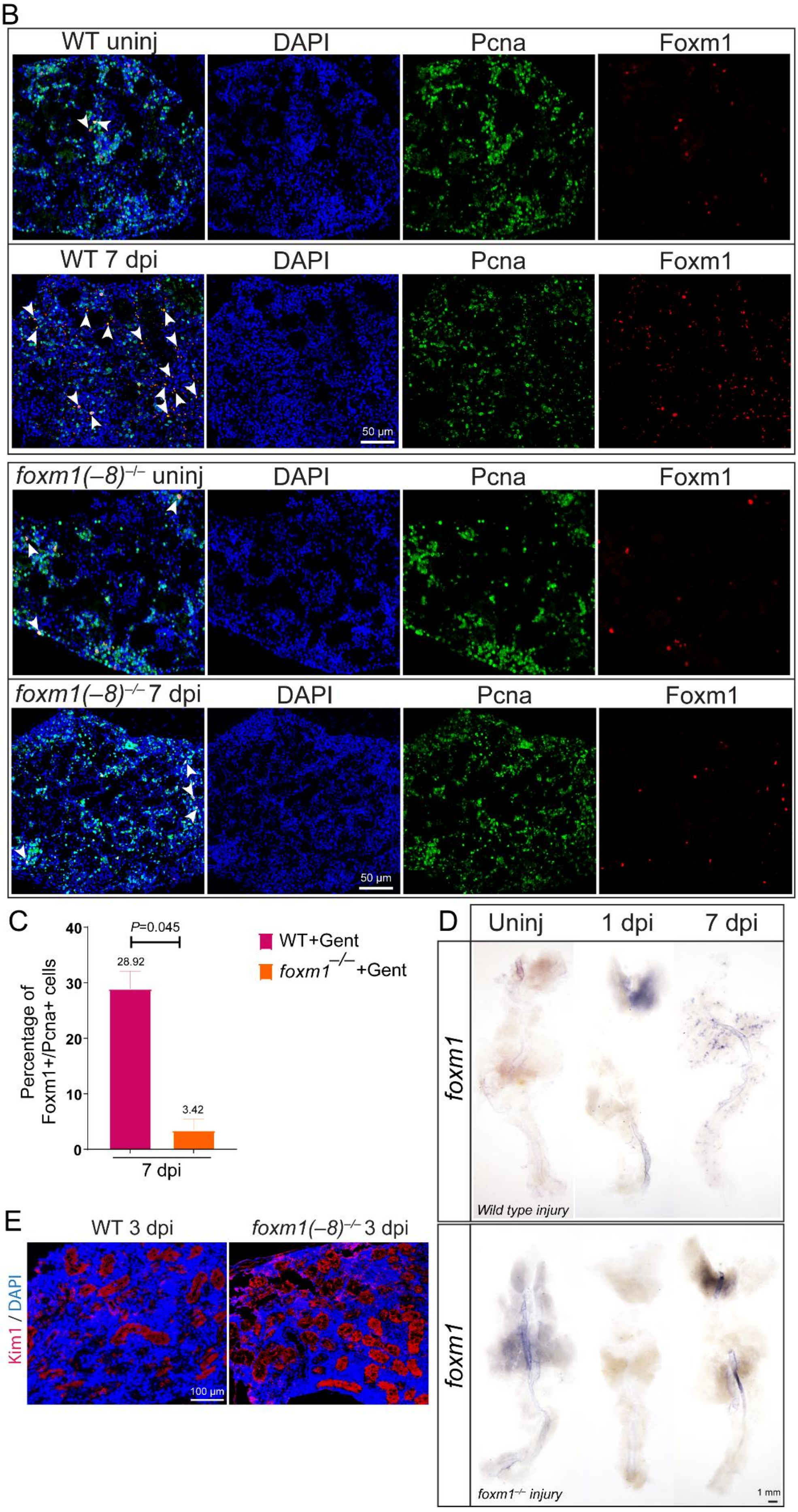

Supplementary Figure S6 *foxm1* is silenced in *foxm1*$^{-/-}$ mutant. (**A**) Sequencing maps of *foxm1*

mutant alleles. (**B**) Images of Foxm1 and Pcna IF in kidneys of wild type and *foxm1*$^{-/-}$ zebrafish at 7 dpi. Few Foxm1 signals were found in mutant and wild type uninjured kidneys, while a sharp decrease of Foxm1 expression in the mutant injured kidneys was found compared to wild type. Arrow heads indicate the Foxm1^{+}/Pcna^{+} cells. (**C**) Quantification of panel B (n = 4-5 different regions of each group) with p values. Gent: gentamicin. IF was repeated three times and representative data are shown. P values for each panel are indicated to show the statistical significance. (**D**) *foxm1 in situ* hybridization of wild type and *foxm1*$^{-/-}$ kidneys at the indicated time points. *foxm1* expression was detected in wild type injured kidneys at 7 dpi and signals in injured mutant kidneys were hardly found. (**E**) Kim1 IF of *foxm1*$^{-/-}$ kidney at 3 dpi after AKI.

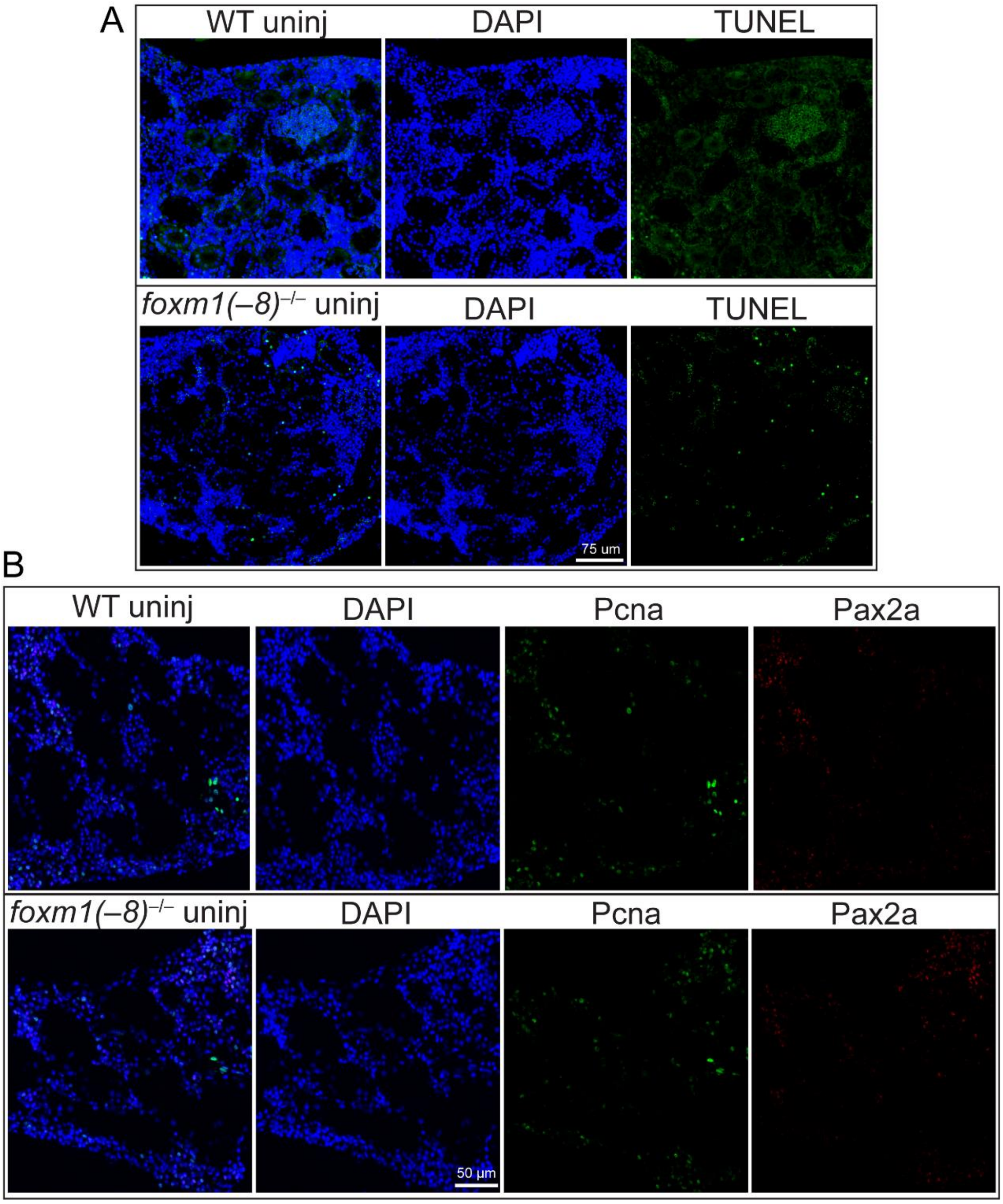

Supplementary Figure S7 No difference in cell apoptosis and proliferation in uninjured *foxm1*$^{-/-}$ kidney. (**A**) TUNEL assay of wild type and *foxm1*$^{-/-}$ uninjured kidneys. (**B**) Double IF images of Pax2a and Pcna from wild type and *foxm1*$^{-/-}$ uninjured kidneys.

A

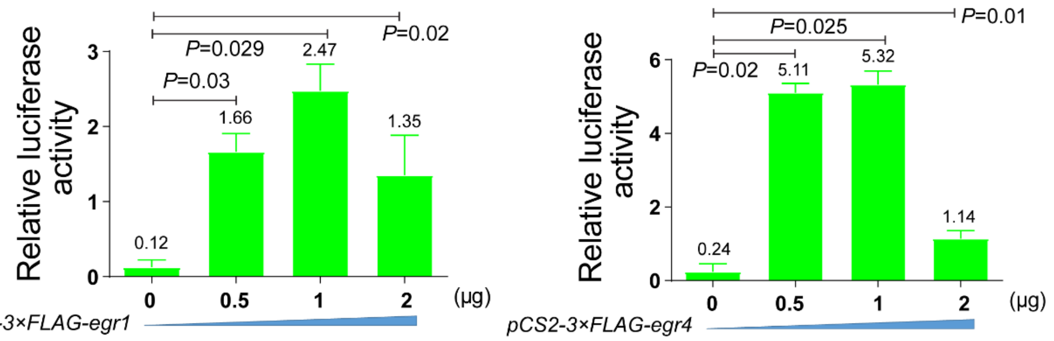

Supplementary Figure S8 *egr1* and *egr4* promote *foxm1* expression under a proper dosage. The changes of luciferase activities under different expressions of FLAG-tagged Egr1/Egr4 in HEK293T cells. ANOVA was used to test the statistical significance of multiple comparisons.

Supplementary materials and methods

Zebrafish maintenance

AB/Tubingen zebrafish were obtained from the China Zebrafish Resource Center, Institute of Hydrobiology, Wuhan, China. Mutants were constructed in AB/Tubingen embryos. Fish strains were raised in a constant temperature water circulation system (28.5□) under a 14-h light/10-h dark cycle. Kidney injury experiments were performed using 5–12 month zebrafish and gender ratios were equal. AB strain zebrafish served as the wild type used as control.

Generation of mutant zebrafish lines by CRISPR/Cas9

Zebrafish mutant alleles were generated by CRISPR/Cas9. Two guide RNAs were designed on exon1 for *egr1* gene; the target sequences were GCTTCCCTCACTCCCCCACGG and CCCCCTTTCTCAACGCCACAG. The target site for *egr4* was designed on exon2; the DNA sequence was CCGCCGTCTCCGCTGACCTAC. A guide RNA was designed on exon 2 for the *foxm1* gene; the sequence was GGGGAAGGAGTGTGGGCCTC.

gRNAs were synthesized using the commercial MEGA Shortscript T7 kit (#AM1354200, Ambion). Two hundred pg purified guide RNA was mixed with 250 pg Cas9 protein (#A36498, Invitrogen), and then the mix was injected into 1-cell stage embryos to knockout the target gene. The injected F0 embryos were raised to adulthood and genotyped for the genetic mosaic mutants. The F0 mutant carriers were crossed with the AB wild type to obtain the F1 mutant fish. F1 adults were outcrossed to obtain F2 homozygous mutants. F2 adults were in-crossed to generate F3 mutants, and in the F3 generation homozygotes were selected using specific primers (Supplementary File 1).

Acute kidney injury by gentamicin treatment

Acute kidney injury was induced in 5-month-old adult wild type and mutant zebrafish (average weight 0.5 g) by the injection of gentamicin (80 mg/kg body weight). A similar volume of PBS was injected, and these zebrafish served as control. Kidneys were removed from anesthetized animals at day 1, 3, 5, 7, 10, 15 post injury (dpi), and 5–10 kidneys or their sections were used for RNA sequencing, qRT-PCR, immunofluorescence (IF), and hematoxylin–eosin (HE) staining. At least 3 experimental repeats were performed.

Bulk RNA-sequencing and heat map analysis

Bulk RNA-sequencing experiments were performed with at least two technical replicates using 8-10 pooled kidneys at each time point, and total RNA was isolated using the TRIzol reagent (ThermoScientific, USA). The RNA library construction and sequencing were performed by the BGI company, Shenzhen, China, using a BGI-500 system.

The RNA-sequencing data were aligned to the zebrafish reference genome using HISTA2. Then, raw counts of all protein coding genes were generated by Feature Counts. Raw counts were normalized to transcript per million (TPM). DESeq2 was performed to calculate differentially expressed genes (DEGs) with |log2(fold change)| > 1 and P-adj < 0.05. ggplot2 was used to make the scatterplot. TPM was used to perform heatmap analysis and the plot was made by pheatmap. DEGs were defined by FDR < 0.05 and log2(fold change) > 1. Gene ontology (GO) analysis for DEGs was performed at https://geneontology.org (accessed on November 23rd, 2023).

Whole mount in situ hybridization (WISH)

The RNA probes were used to detect the expression of *slc20a1a, trpm7, slc12a1, slc12a3, lhx1a, egr1, egr4,* and *foxm1*. The primers used for probe synthesis are listed in Supplementary File 1. Templates for probe synthesis were amplified by PCR from zebrafish embryonic cDNA using primers containing the T7 RNA polymerase promoter sequence. Purified PCR products were transcribed *in vitro* and labeled using digoxigenin (DIG) RNA labeling Kit (#11277073910, Roche) according to the manufacturer's protocol. RNA probes were purified using mini-Quick Spin RNA Columns (#11814397001, Roche) and stored at −80°C in deionized formamide. WISH was performed according to the standard protocol.

PCR analysis

qRT-PCR was used to detect the expression of genes of interest. The primers are listed in Supplementary File 1. Briefly, total RNA was extracted from 8–0 pooled zebrafish kidneys using the TRIzol kit (Invitrogen, USA). At least three replicates were used, with each replicate composed of 8–10 kidneys. After cDNA synthesis from total RNA, PCR was performed in a Bio-Rad instrument. qRT-PCR data were processed by GraphPad Prism 9.

Nephron function evaluation by Dextran-FITC absorbance

Dextran-FITC (#R-FD-003, RuiXi Bio) was intraperitoneally injected into zebrafish, the kidneys were collected after 8–12 h, and fixed with 4% PFA for 4 h at room temperature. Kidneys were washed three times with PBS and dehydrated in sucrose solution (15% sucrose/PBS, 30% sucrose/PBS) for 2 h at 4□. Kidneys were embedded in Tissue-Tek O.C.T compound (#4583, Sakura) and cut into 30 μm-thick slices on a cryostat. Slides containing kidney sections were dried at room temperature for 30 min and washed with PBS 3 times for 5 min at room temperature. Slides were mounted using anti-fade mounting medium (#SI103-02, SEVEN). Mounted slides were imaged using a Leica Confocal Microscope (TCS SP8 STED, Germany). The detailed procedure for performing Dextran labeling PTs was previously described.

Immunofluorescence (IF) experiments

Anesthetized zebrafish were fixed in 4% PFA, and kidneys were detached from the body wall. Kidneys were washed three times with PBS and dehydrated in sucrose solution (15% sucrose/PBS, 30% sucrose/PBS) for 2 h at 4□. Kidneys were embedded in Tissue-Tek O.C.T compound (#4583, Sakura) and cut into 30 μm-thick slices on a cryostat. Slides containing kidney sections were dried at room temperature for 30 min and washed with PBS 3 times for 5 min at room temperature. Sections on slides were blocked using 5% normal bovine serum (#A2153, Sigma Life Science) in PBS + 0.1% Triton-X100 (#V900502, VETEC) for 2 h. After washing, the following primary antibodies (1:500) were added: anti-Pax2a (#ab229318, abcam), anti-Kim1 (#AF1817, R&D system), anti-Pcna (#GTX124496, GeneTex), and anti-Foxm1 (#AF7860, Affinity Biosciences LTD). Slides were washed with PBS 3 times for 5 min, followed by the incubation with the secondary antibody Alexa 488 or Alexa 555 (#A11008, #A21428, Invitrogen) (1:500) for 2 h. Slides were counter-stained with DAPI (#D9542, Sigma, USA) and/or Lotus tetragonolobus lectin (LTL, #FL-1321, Vector Laboratories) in 1×PBS at room temperature for 2 h. Slides were washed 3 times with PBS, mounted using anti-fade mounting medium (#SI103-02, SEVEN), and imaged using a Leica Confocal Microscope (TCS SP8 STED, Germany).

ChIP experiments

ChIP experiments were performed according to the Agilent Mammalian ChIP-on-chip Manual as described. Briefly, embryos were processed to single cells and fixed with 1% formaldehyde for 10 min at room temperature. The reaction was stopped using 0.125 M glycine for 5 min under rotation. The fixed chromatin was sonicated to an average of 500–1000 bp (for ChIP-qPCR) using the S2 Covaris Sonication System (USA) according to the manual. Then, Triton X-100 was added into the sonicated chromatin solution to a final concentration of 0.1%. After centrifugation, 50 μl supernatant was used as input. The remaining chromatin solution was incubated with Dynabeads previously coupled with 5 μg ChIP grade antibody (#F1804, SIGMA) overnight at 4°C under rotation. The next day, the complexes were washed 7 times with the wash buffer and reverse cross-linked overnight at 65°C. DNA was extracted by hydroxybenzene-chloroform-isoamyl alcohol and purified by a Phase Lock Gel (Tiangen, China). The enriched DNA by antibody was dissolved in 100 μl distilled water. Quantitative real-time PCR (qRT-PCR) was performed using a Bio-Rad instrument. The enrichment was calculated relative to the amount of input as described. All experiments were repeated at least two times. The relative gene expression was calculated using the $2^{-\Delta\Delta Ct}$ method. Statistical analysis was performed using a paired *t*-test. Results are expressed as mean ± S.D.

Luciferase assay

A 2926-bp *foxm1* promoter was amplified using the following primer pairs: forward primer GCTCGCTAGCCTCGACAGAGGAGCCACCGATCGC, reverse primer CCGGATTGCCAAGCTGTCTCCTTGGGCTCTCCC. The DNA fragment was cloned into the luciferase reporter-containing vector *pGL4-Luci* (Promega, Madison, USA). Luciferase assay was performed using the Dual-Luciferase Reporter Assay System (Promega, Madison, WI) and MiniLumat LB9506 (Berthold, Germany). Luciferase reporter assay in HEK293T was performed using 13105 cells seeded in 24-well plates in DMEM medium containing 10% FBS (#FS101-02, Transgen Biotech). Cells were transfected with the indicated vectors using Hieff trans TM Liposomal Transfection reagent (#40802ES03, Yeasen).
